# Size-Dependent Reduction Kinetics of Iron Oxides in Single and Mixed Mineral Systems

**DOI:** 10.1021/acs.est.4c08032

**Published:** 2025-01-29

**Authors:** Xiyang Xu, Muammar Mansor, Guoxiang Li, Tsz Ho Chiu, Stefan B. Haderlein, Andreas Kappler, Prachi Joshi

**Affiliations:** † Geomicrobiology, Department of Geosciences, University of Tübingen, 72076 Tübingen, Germany; ‡ Environmental Chemistry and Mineralogy, Department of Geosciences, University of Tübingen, 72076 Tübingen, Germany; § Cluster of Excellence: EXC 2124: Controlling Microbes to Fight Infection, 72076 Tübingen, Germany

**Keywords:** iron minerals, nanoparticles, microbial reduction, mediated electrochemical reduction, reduction kinetics

## Abstract

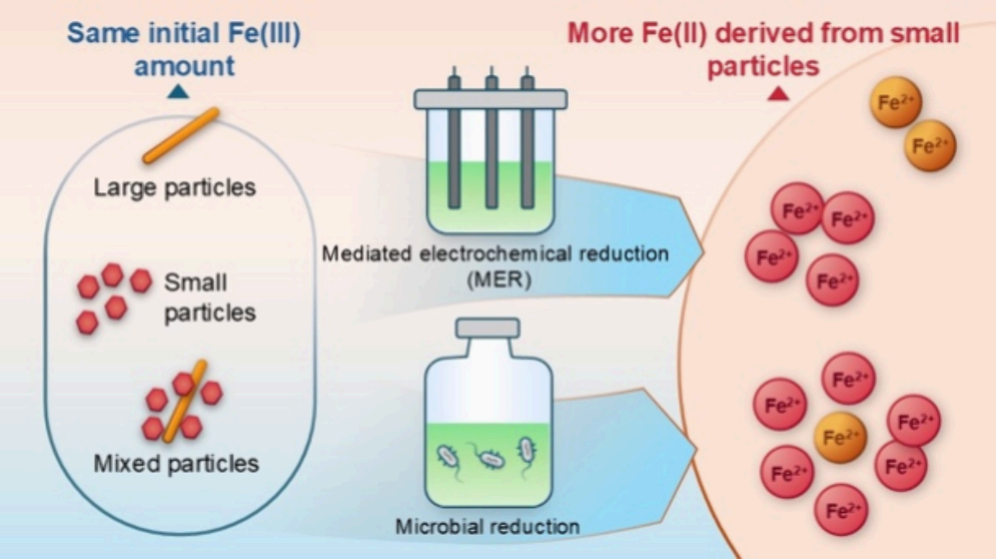

Iron(III) (oxyhydr)oxide minerals with varying particle sizes commonly coexist in natural environments and are susceptible to both chemical and microbial reduction, affecting the fate and mobility of trace elements, nutrients, and pollutants. The size-dependent reduction behavior of iron (oxyhydr)oxides in single and mixed mineral systems remains poorly understood. In this study, we used microbial and mediated electrochemical reduction approaches to investigate the reduction kinetics and extents of goethite and hematite. We found that small particles were preferentially reduced relative to their large counterparts in single and mixed mineral systems regardless of microbial or electrochemical treatments, which is attributed to the combined effect of higher thermodynamic favorability and greater surface availability. In mixed mineral systems, small particles were reduced slightly faster, whereas large particles were reduced notably slower and less extensively than solely predicted from single mineral systems. Specifically, when reduced alone, small particles showed Fe(III) reduction rate constants that were 1.5- to 3.6-fold higher than large particles, while when reduced together, the reduction rate constants for small particles were 6- to 21-fold higher than the rate constants for large particles. These collective findings provide new insights into the pivotal role of nanoparticulate iron (oxyhydr)oxides in environmental redox reactions.

## Abstract 2

Small particles of iron(III) (oxyhydr)oxide were reduced faster and more extensively than larger counterparts in single and mixed mineral systems.

## Introduction

Iron(III) (oxyhydr)oxide minerals, such as goethite (α-FeOOH) and hematite (α-Fe_2_O_3_), are ubiquitous in soils and sediments,^1−3^ where they span a continuum of sizes, from nanometer to the millimeter scale.^2,4^ Nanoparticles behave remarkably differently from their larger counterparts, resulting in enhanced roles in the biogeochemical cycling and fate of nutrients, trace elements, and contaminants via redox reactions.^5−11^ For instance, nanoparticulate iron (oxyhydr)oxides can act as terminal electron acceptors in anaerobic microbial respiration,^12−15^ resulting in their dissolution and the subsequent release of associated trace elements.^16,17^

The significance of iron redox reactions has led to extensive research over the past decades into the susceptibility of iron (oxyhydr)oxides toward microbially and chemically driven reduction processes. The extents and rates of Fe(III) mineral reduction have been suggested to depend on the characteristics of the minerals such as particle size, morphology, crystallinity, reactive surface area, vacancies, solubility, dissolution, thermodynamic properties as well as cell–mineral attachment, secondary biomineralization, and presence of electron shuttles.^14,18−30^ Notably, Roden (2003) observed a strong linear correlation between the initial rate of microbial Fe(III) reduction and mineral surface area and suggested that microbial Fe(III) mineral reduction was not significantly influenced by the thermodynamic properties of the iron (oxyhydr)oxides.^20^ In contrast, Bonneville et al. (2004, 2006) proposed that solubility and coverage of the cell surface by hematite nanoparticles were rate-controlling factors in both biotic and abiotic reduction of the hematite.^25,26^ Yan et al. (2008) suggested that the reduction rates of nanohematite were likely to be proportional to the cell–mineral contact area and not to the total surface area.^27^ In addition, Aeppli et al. (2018, 2022) postulated that thermodynamics controlled goethite and hematite reduction kinetics in mediated electrochemical experiments.^28,29^ In these studies as well as other past work, identifying effects of particle size on reduction rates and extents has been elusive because comparative experiments typically investigated either different minerals or the same mineral with one or two properties that were varied. However, evaluating these variables independently is difficult, as they are all related. Therefore, accurately determining the size effects on reduction kinetics of nanoparticles requires systematic investigation of the same mineral with all relevant explanatory factors.

While abundant literature has described the reduction of individual iron (oxyhydr)oxides, these minerals often coexist as mixtures of mineral phases^1,31−35^ and varying particle sizes in the environment.^36,37^ For instance, coexisting goethite and hematite have been reported in ocean sediments,^32^ lacustrine sediments,^33^ loess-paleosol sequences,^34^ and soils.^31,35^ Although the coexistence of minerals in the environment is expected, there have been only a few studies examining how coexistence affects overall reactivity.^38−41^ For example, Notini et al. (2019) observed that goethite with defects displayed an initial microbial Fe(III) reduction rate about 6-fold higher than goethite with fewer defects, when these minerals were present together.^38^ Hua et al. (2022) studied microbial Fe(III) reduction with respect to the coexistence of hematite with multiple facets, showing that hematite nanoplates were reduced 1.8 times more and 1.6 times faster than hematite nanorods.^39^ Other studies have looked at processes other than reduction in mixed systems: Notini et al. (2023) demonstrated that the coexistence of goethite promoted the transformation of ferrihydrite into goethite.^40^ Higher ratios of goethite to ferrihydrite have been shown to promote heteroaggregation, which may impact their combined reactivity.^41^ However, no studies to date have examined the reduction behavior of iron (oxyhydr)oxides in mixed mineral systems with varying particle sizes.

In the present study, the reduction kinetics and extent of iron (oxyhydr)oxides in single and mixed mineral systems were systematically investigated using microbial and mediated electrochemical reduction (MER) approaches. The work had two specific objectives. (i) To evaluate the impact of mineral-specific related variables on their reduction kinetics and extents within single mineral systems through redundancy analysis (RDA) and Pearson correlation analysis. Here, we utilized the unique capability of MER to adjust the thermodynamic boundary conditions in order to study the thermodynamic driving force of iron (oxyhydr)oxide reduction, complementary to microbial reduction.^42,43^ (ii) To determine the size preferential reduction behavior of iron (oxyhydr)oxides within environmentally relevant mixed mineral systems. We employed Fe isotope-labeled minerals to track the origin of reduced Fe in mixed mineral systems during microbial reduction processes. The findings of this research enhance our understanding of the reduction susceptibility of iron (oxyhydr)oxides with varying particle sizes in the environments.

## Materials and Methods

### Iron (oxyhydr)oxide Synthesis and Characterization

Goethite and hematite with varied particle sizes were synthesized based on previous methods.^44−46^ The synthesized minerals are termed Gt_2000, Gt_90, Gt_30, Hm_300, Hm_40, and Hm_8 based on their particle size. We synthesized a second set of iron (oxyhydr)oxides for mixed mineral experiments, which included naturally abundant Fe minerals (referred to as ^NA^mineral, containing 5.82% of ^54^Fe) and ^56^Fe-enriched Fe minerals (referred to as ^56^mineral, containing negligible ^54^Fe, prepared from ^56^Fe-enriched Fe^0^ (Isoflex, 99.94% purity)). The purities of the synthesized iron (oxyhydr)oxides were determined using micro-X-ray diffraction (μ-XRD) and ^57^Fe Mössbauer spectroscopy. The morphologies and particle size distributions of the iron (oxyhydr)oxides were characterized using scanning electron microscopy (SEM) and transmission electron microscopy (TEM). The BET surface area was determined by N_2_ sorption at 77 K. The zeta potentials and aggregation sizes of the iron (oxyhydr)oxides were measured by dynamic light scattering (DLS, Malvern Zetasizer Nano ZS).^47^ Details regarding the synthesis and characterization are provided in the SI, Section S1.

### Mediated Electrochemical Fe(III) Reduction

To study reduction kinetics and extents of the iron (oxyhydr)oxides at different applied reduction potentials (*E*_H_^MER^) as a function of particle size, mediated electrochemical reduction (MER) experiments were conducted under anoxic conditions inside a glovebox (100% N_2_ atmosphere, O_2_ < 2 ppm). The electrochemical setup consisted of 9 mL glassy carbon cells as the working electrode (WE), Ag/AgCl as the reference electrode, and a platinum wire as the counter electrode. The cells were controlled by eight-channel potentiostats (model 1000B, CH Instruments). The *E*_H_^MER^ was measured against Ag/AgCl reference electrodes, but we herein report values versus standard hydrogen electrode (SHE). Throughout the experiments, the solution in each WE cell was stirred continuously with a glass-coated stir bar.

To perform the MER, we first filled the WE cells to 6.5 mL with buffer solution (HEPES, 10 mM, pH 7) with NaCl as an electrolyte (100 mM) and then applied a defined and constant *E*_H_^MER^ to the WE. After the background current had stabilized at a low value (<4 μA), mediator (10 mM) was added to the cell: 9,10-anthraquinone-2,6-disulfonic acid disodium salt (AQDS, *E*_H_^0^ = −0.18 V)^48^ at *E*_H_^MER^ = −0.25 V, diquat (DQ, *E*_H_^0^ = −0.35 V)^49^ at *E*_H_^MER^ = −0.35 V, and 4,4′-bipyridinium-1,1′-bis(2-ethylsulfonate) (ZiV, *E*_H_^0^ = −0.41 V)^43^ at *E*_H_^MER^ = −0.53 V. Each mediator was transferred into the WE cell in duplicate additions, followed by triplicate spikes of the iron (oxyhydr)oxides and finally duplicate additions of the mediator (SI, Figure S6). The intervals between spikes were long enough to allow the current to stabilize (<4 μA). The experiments were designed such that the concentration of reduced mediator (833 μM) was approximately 6 times higher than the iron(III) concentration (138 μM) after the first addition of the iron (oxyhydr)oxide suspension. At this ratio, maximum iron (oxyhydr)oxide reduction rates have been reported to be independent of the mediator concentration.^28^

The reductive current peaks in response to the four mediator additions were utilized to determine the rate constants of the mediator reduction in individual cells. These rate constants were subsequently employed to normalize the iron (oxyhydr)oxide reduction rate constants calculated from individual cells (see the Data Analysis section below).

### Microbial Fe(III) Reduction

To study the kinetics and extents of microbial reduction of iron (oxyhydr)oxides, we performed cell suspension experiments using the fermentative Fe(III)-reducer *Shewanella oneidensis* MR-1 (details of incubation in SI, Section S3). Batch microbial Fe(III) reduction experiments were carried out in triplicates using serum bottles filled with 25 mL of anoxic buffer solution (HEPES 15 mM, pH 7). The HEPES buffer was chosen to avoid interactions with dissolved Fe(II) and minimal effect on nanoparticle dissolution or etching.^50^ Sodium lactate (5 mM) and iron (oxyhydr)oxides (10 mM) were used as electron donors and acceptors, respectively, and AQDS (20 μM) was employed as an electron shuttle to mimic natural redox-active mediators such as NOM and EPS. Washed cells with a concentration of 5 × 10^8^ cells/mL were added into the reactors. The cell number was determined using optical density (OD) measurements. Serum bottles were placed (stationary) at 28 °C in the dark for 7 days. An aliquot of the suspension (0.5 mL) was periodically removed (at 3, 6, 12, 25, 35, 50, and 75 h) inside the N_2_-filled glovebox, with the aqueous and solid phases separated by centrifugation (12,100*g*, 15 min). The solids were sequentially extracted using 0.5 M HCl (30 min at 25 °C in the glovebox) and 6 M HCl (8 h at 60 °C in the glovebox). Based on the above sampling, we quantified the concentration of aqueous, adsorbed, and total Fe(II) using the ferrozine assay.^51,52^

### Microbial Fe(III) Reduction of Isotopically Labeled Mixed Minerals

To study size preferential microbial reduction in environmentally relevant complex systems with varying particle sizes and mineral types, we designed three experimental treatments: a mix of (1) ^56^Gt_2000 (Fe concentration of 5 mM) and ^NA^Gt_90 (5 mM), (2) ^56^Hm_40 (3.5 mM) and ^NA^Hm_8 (6.5 mM), and (3) ^NA^Gt_90 (4.5 mM) and ^56^Hm_8 (5.5 mM). The concentrations were chosen so as to approximate their number-based size distribution based on Pareto’s law, which shows that particle number concentrations increase logarithmically with each order of magnitude decrease in particle size.^36,37^ Detailed calculations are provided in the SI, Section S5. The use of mixtures of ^NA^mineral and ^56^mineral allowed us to utilize ^54^Fe and ^56^Fe to track the origin of the reduced iron.

Experiments were performed as described above for microbial Fe(III) reduction. The isotopic compositions of obtained aqueous phase and sequentially HCl-extracted phases were determined by inductively coupled plasma mass spectrometry (ICP-MS, Agilent 7900), with standard deviations <10% for both ^54^Fe and ^56^Fe.

### Data Analysis

The number of electrons accepted by the iron (oxyhydr)oxides in MER experiments, *q* [mol_e_^–^], was quantified by integration of the reductive current in response to iron (oxyhydr)oxide spikes, according to eq 1.^49^
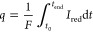
1where *I*_red_ [A] is the background-subtracted reductive current, *F* is the Faraday constant, and *t*_*o*_ and *t*_end_ [s] represent the initial and end integration boundaries for each current peak. Background subtraction and peak area integration were manually performed using Origin 2021 (9.8).

(a) The observed rate constants for MER reduction, *k*_obs_ [s^–1^], were determined from the current responses to the first addition of iron (oxyhydr)oxides in MER experiments, according to eq 2.^43^
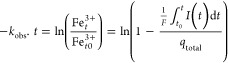
2where *t*_0_ and *t* [s] are the time of iron (oxyhydr)oxide addition and the time during (oxyhydr)oxide reduction, respectively; Fe_*t*_^3+^/Fe_*t*0_^3+^ denotes the fraction of the remaining ferric iron at time *t* relative to initially added ferric iron in the WE cell; and *I*(*t*) [A] is the background-subtracted reductive current. *k*_obs_ was determined from the slope of a linear regression line fitted to the reductive current response plotted as ln(Fe_*t*_^3+^/Fe_*t*0_^3+^) versus time, where data were collected from the time point at maximum reductive current to the time point at 90% of final reduction extent. We considered only the first addition due to its greater reduction extent and lower activities of Fe^2+^ relative to the second and third additions.

The reduction rate constants for the mediator, *k*_obs_^med^ [s^–1^], were determined from the reductive peak in response to mediator additions using a modified eq 2 (i.e., ln(med_red_(*t*)/med_red_(*t*_0_)) instead of ln(Fe_*t*_^3+^/Fe_*t*0_^3+^)). *k*_obs_^med^ was utilized to normalize differences between separate WE cells, which might result from the stirring speed and depth of the counter electrode in the cell (eq 3).
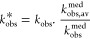
3where *k*_obs_^*^ [s^–1^] is the corrected observed rate constant for iron (oxyhydr)oxide reduction and *k*_obs,av_^med^ [s^–1^] is the averaged mediator reduction rate constants for each mediator across all experiments.

(b) The microbial reduction rate was calculated based on pseudo-first-order kinetics. The rate constant, *k*′, was determined from the slope of a linear regression plotted as ln(Fe_*t*_^3+^/Fe_total_^3+^) versus time, according to eq 4.^18^
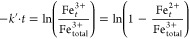
4

where *t* [h] is the time, Fe_t_^3+^/Fe_total_^3+^ denotes fraction of the remaining ferric iron at time t relative to initially added ferric iron, and Fe_*t*_^2+^ is the ferrous iron concentration at time *t*, which was determined following 6 M HCl extraction.

### Thermodynamic Calculations

(a) Standard reduction potential (*E*_H_^0^) of iron (oxyhydr)oxides was calculated from standard Gibbs free energy of reaction (Δ*G*_*r*x*n*_^0^). The Gibbs free energy of nanoparticle formation Δ*G*_*f*,*i*_^0^ was calculated based on the published values for bulk minerals and corrected for increased surface energy of nanoparticles through published mineral-specific enthalpies of hydrated surfaces (Δ*H*_*s*_^*h*^) and molar volumes (*S*_*m*_), assuming geometric surface area (SA) and volume (*V*), according to eqs 5 and 6.^53−55^
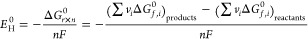
5
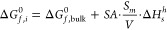
6where Δ*G*_*f*,bulk_^0^ [kJ/mol] is the Gibbs free energy of bulk mineral formation of reactants and products, *n* denotes the number of transferred electrons, and *v*_*i*_ represents the stoichiometric coefficient of species *i* in the balanced chemical equations (SI, Section S4).
(b) Reaction free energies, Δ_*r*_*G* [kJ/mol], in MER experiments were calculated from differences in reduction potential between iron (oxyhydr)oxide and applied potential, according to eq 7.
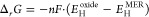
7
  *E*_H_^oxide^ was calculated from the Nernst equation according to eq 8.
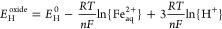
8where *R* is the ideal gas constant, *T* is the absolute temperature, and {Fe_aq_^2+^} denotes the activity of aqueous ferrous iron calculated from Davies equation.^56^ According to eqs 7 and 8, Δ_*r*_*G* is dependent on the dissolved ferrous iron activity. We herein calculated Δ_*r*_*G* for selected {Fe_aq_^2+^} that corresponded to the time of the maximum rate of electron transfer, when data collection began to calculate rate constant *k*_obs_. A detailed description of the Δ_*r*_*G* calculations is provided in the SI, Section S4.

### Statistical Analysis

Detrended correspondence analysis (DCA) and redundancy analysis (RDA) were conducted using CANOCO 5 software (Microcomputer Power Co., USA), and Pearson correlation was performed with Origin 2021 (9.8) (OriginLab Co., USA). RDA, widely used in ecology for direct gradient analysis, can evaluate the relationship between a set of explanatory and the target variables, irrespective of whether the variables are related.^57^ Before RDA, detrended correspondence analysis (DCA) was performed to determine whether the data sets were appropriate for RDA. If the gradient lengths of the four axes calculated from DCA are less than 3, then RDA was applicable. The DCA results for the variables in both the MER and microbial reduction experiments indicate gradient lengths of 0.18 and 0.28, respectively, suggesting that the data set was suitable for RDA.

## Results and Discussion

### Characterization of Goethite and Hematite Nanoparticles

To study the size-dependent reduction kinetics and extents of goethite and hematite in single and mixed mineral systems, we synthesized goethite and hematite with a range of different sizes (30–2000 nm for goethite and 8–300 nm for hematite). The μ-XRD patterns (SI, Figure S1) and Mössbauer spectra (SI, Figure S2 and Table S1) indicated pure goethite and hematite. Widths of the reflections in XRD patterns increased with decreasing sizes, indicating a lower crystallinity. Additionally, differences in Mössbauer fitting parameters showed small variations in magnetic properties of samples. Based on the average length of goethite particles and mean diameters of hematite particles in TEM or SEM images (SI, Figures S3–S5), the minerals are referred to heretofore as Gt_2000, Gt_90, Gt_30, Hm_300, Hm_40, and Hm_8.

### Mediated Electrochemical Fe(III) Reduction of Goethite and Hematite

**Figure 1:**
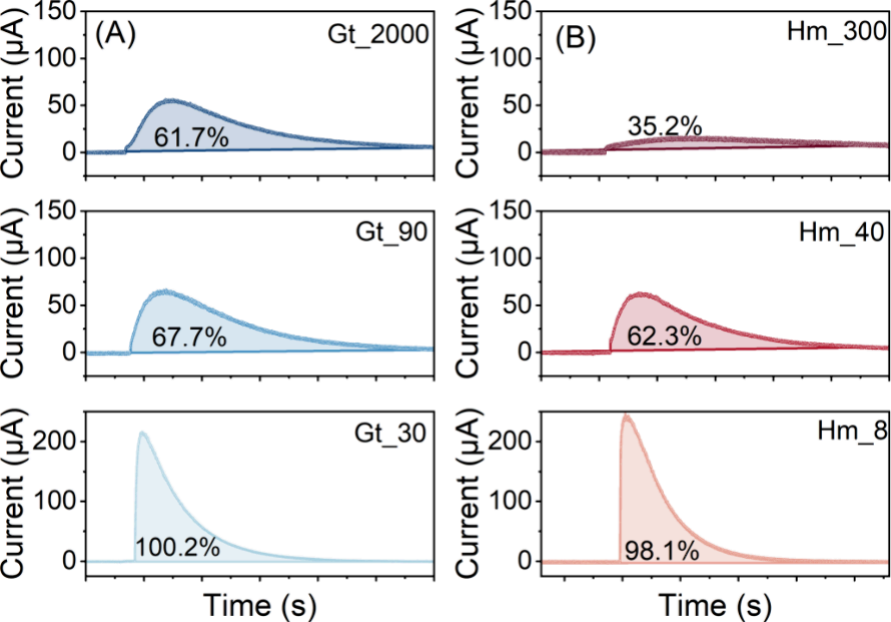
Exemplary reductive current responses to the addition of goethite (Gt) (A) and hematite (Hm) (B) to electrochemical cells at pH 7.0 and *E*_H_^MER^ = −0.35 V. Gt_2000, Gt_90, and Gt_30 denote goethite sizes of 2000, 90, and 30 nm, respectively. Hm_300, Hm_40, and Hm_8 denote hematite sizes of 300, 40, and 8 nm, respectively. Colored areas represent the number of electrons transferred by peak integration, and the percentage values represent the total Fe(III) reduction extent.

To investigate size-dependent reduction of goethite and hematite via a mediated electrochemical approach, we measured reductive current responses to additions of these two iron (oxyhydr)oxides in electrochemical cells at different applied potentials that contained an electron transfer mediator (*E*_H_^MER^ = −0.53, −0.35, −0.25 V vs SHE at pH 7.0). Figure 1 shows reductive current responses to additions of goethite (Figure 1 A) and hematite (Figure 1 B) at *E*_H_^MER^ = −0.35 V. We observed that decreasing particle sizes resulted in increasing heights and areas of reductive current peaks for goethite and hematite. Similar trends were observed at other *E*_H_^MER^ values (−0.53 and −0.25 V) (SI, Figures S2, S7–S9).

We determined the reduction extents and rates from the reductive current responses. At *E*_H_^MER^ = −0.53 V, the reduction extents exceeded 90% for both goethite and hematite regardless of particle size (SI, Table S2), consistent with the thermodynamic favorability of complete iron (oxyhydr)oxide reduction at this low *E*_H_.^28^ By contrast, the reduction extents were lower with increasing (i.e., less negative) *E*_H_^MER^ of −0.35 and −0.25 V. Smaller-sized minerals were especially more susceptible to reduction compared to their larger counterparts or when subjected to higher applied *E*_H_^MER^ conditions. For instance, Gt_2000 was reduced by 61.7% at −0.35 V, while Gt_30 was reduced completely (100.2%). This increased susceptibility is likely due to the higher redox potential of the smaller particles.

In terms of reduction rate, we chose to present corrected observed rate constants,^28,43^ (*k*_obs_^*^), for goethite and hematite at *E*_H_^MER^ of −0.35 and −0.53 V, at which all minerals can be reduced to comparatively high extents (from 35.2 to 100.2%). We found that *k*_obs_^*^ values clearly increased with decreasing particle sizes irrespective of *E*_H_^MER^ (Table 1). This demonstrated that the reduction rates were strongly dependent on the mineral particle size. In addition, we calculated the *k*_obs,av_^med^ for different mediator species used in MER experiments (*k*_obs,av_^med^ = 11.19 ± 0.52 h^–1^ for DQ at −0.35 V and 7.75 ± 0.26 h^–1^ for ZiV at −0.53 V). At *E*_H_^MER^ = −0.53 V, the *k*_obs_^*^ for Gt_30 and Hm_8 reduction (8.61 and 8.00 h^–1^, respectively; Table 1) was slightly higher than the *k*_obs,av_^med^ (7.75 h^–1^). This indicated that the rates of mediator rereduction masked the rates of electron transfer from the reduced mediator to those iron minerals. Consequently, the observed reduction rate constants for Gt_30 and Hm_8 likely do not represent their maximum reduction rate, which could be higher in a case where the mediator re-reduction was faster. Conversely, the rest of the reduction rate constants for the (oxyhydr)oxides were lower than the *k*_obs,av_^med^, which denoted that the observed reduction rates were controlled by the rates of electron transfer from the reduced mediator to the minerals instead of the redox cycling of the mediators.

**Table 1:** Reduction Rate Constants of Goethite and Hematite as a Function of the Particle Size

mineral	microbial reduction^a^	mediated electrochemical reduction	
		*E*_H_^MER^ = −0.35 V^b^	*E*_H_^MER^ = −0.53 V^c^
	h^–1^ (×10^–3^)	h^–1^	h^–1^
Gt_2000	1.45 ± 0.03	2.89 ± 0.26	3.95
Gt_90	2.21 ± 0.19	3.38 ± 0.23	4.67
Gt_30	6.05 ± 0.17	8.19 ± 0.16	8.61
Hm_300	0.48 ± 0.02	1.60 ± 0.09	2.20
Hm_40	1.74 ± 0.10	3.55 ± 0.08	4.93
Hm_8	6.20 ± 0.36	7.63 ± 0.31	8.00

a Microbial reduction was carried out in triplicates. Values represent the mean ± standard deviation for a given mineral.

b For all iron (oxyhydr)oxides at *E*_H_^MER^ = −0.35 V, duplicate MER experiments were carried out simultaneously in two separate electrochemical cells. Reported rate constants are the average of the duplicate measurements (±depict deviation of single measurements from the mean).

c For all iron (oxyhydr)oxides at *E*_H_^MER^ = −0.53 V, MER experiments were performed in individual cells.

In the case of electrochemical reduction, we could quantitatively compare the relative thermodynamic driving force. We first calculated theoretical *E*_H_ values, which ranged from +765 to +832 mV (for goethite) and from +764 mV to +863 mV (for hematite) (eqs 5 and 6, SI, Table S4).^53−55^ For Hm_40 and Hm_8, the *E*_H_ values (+781 mV, + 863 mV) were similar to previously reported values for similarly sized particles (+773 ± 6 and +823 ± 6 mV).^58^ We then calculated Δ_*r*_*G*, which yielded values from −49.3 to −60.0 kJ/mol for all goethite and hematite at *E*_H_^MER^ = −0.35 V and from −55.2 to −66.5 kJ/mol at *E*_H_^MER^ = −0.53 V (SI, Section S4). At more negative Δ_*r*_*G* values and more positive *E*_H_ values, the reduction of iron (oxyhydr)oxide was much more thermodynamically favorable, thus leading to a faster and more extensive reduction.

### Microbial Fe(III) Reduction of Goethite and Hematite

**Figure 2:**
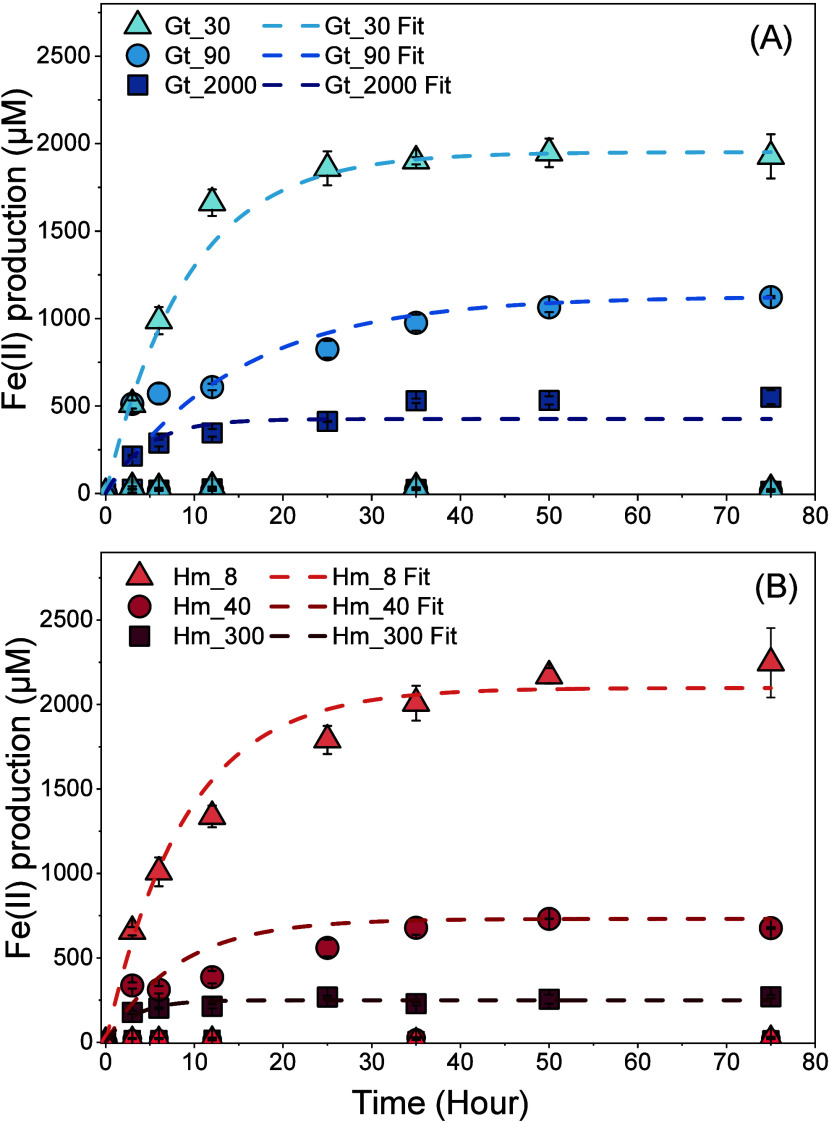
Total Fe(II) production by microbial Fe(III) reduction of goethite (blue) (A) and hematite (red) (B) as a function of particle size. Dashed lines denote the fit based on pseudo-first order kinetics. Symbols correspond to the average of biological triplicates, and the error bars indicate standard deviations. Experimental conditions: 10 mM Fe(III) of goethite/hematite; 5 × 10^8^ cells mL^–1^*S. oneidensis* MR-1 (no cells in abiotic controls); 20 μM AQDS; pH 7.

To evaluate size-dependent susceptibility of goethite and hematite toward microbial Fe(III) reduction, we tracked the production of Fe(II) over time (Figure 2). With decreasing particle sizes, reduction extents varied from 6.3 to 16.8% for goethite and from 2.7 to 21.7% for hematite (SI, Table S2). The results indicate that smaller-sized minerals were more susceptible toward microbial Fe(III) reduction, which is consistent with mediated electrochemical reduction.

Rate constants (*k’*) for microbial Fe(III) reduction were derived using pseudo-first-order kinetic model (Figure 2), which provided a highly reliable fit to the experimental data (*R*^2^ > 0.99 for both samples). Rate constants increased with decreasing particle sizes for both goethite and hematite (Table 1), which is consistent with the trend observed in the mediated electrochemical experiments. Microbial reduction rates were much more sensitive to size compared with mediated electrochemical reduction. For example, Gt_30 was reduced 4.2× faster than Gt_2000 via microbial reduction, compared to only 2.2–2.8× increase for MER (at *E*_H_^MER^ = −0.35 or −0.53 V). The same is true and more pronounced when comparing Hm_8 and Hm_300 (12.9× for microbial and only 3.6–4.8× for electrochemical reduction). To test whether this observed trend is influenced by the presence of AQDS as an electron shuttle, we also performed microbial experiments in the absence of AQDS. We observed a faster and more extensive reduction of smaller particles, although overall slower than that in the presence of AQDS (Figure S11, Table S3). These findings indicate that microbial reduction is more strongly influenced by the mineral size than the presence of electron shuttles.

### Determining Impacts of Related Variables on Mineral Reducibility via MER and Microbial Reduction

The extents and rates of Fe(III) mineral reduction have been suggested to be influenced by a variety of related factors,^14,18−23^ making it challenging to evaluate these variables independently. For example, crystallite size influences both the surface areas and the thermodynamics (i.e., Δ_*r*_*G* and *E*_H_ of the mineral).^54^ Crystallite size and particle concentration also affect the degree and type of aggregation (e.g., pore size), which then further affects the reactive surface areas.^59,60^ In addition, different techniques are able to measure different types of sizes^61^ (e.g., crystallite size via XRD, particle size via electron microscopy, and aggregation size via DLS) and surface areas^62^ (e.g., measured via BET or calculated from shapes observed under electron microscopy). Among these variables, surface area and thermodynamics have been strongly proposed to control mineral reduction processes.^20,28,29^ To probe the individual and combined impact of these two variables, we therefore performed a correlation analysis between rate constant normalized using BET-derived surface area and free reaction energy (Δ_*r*_*G*) or standard redox potential (*E*_H_^0^) (SI, Figure S12). Our results indicated that neither variable alone nor their combination can fully account for the observed variations in reduction rate constants. Thus, a data analysis technique that incorporates these related variables (also called “explanatory variables”) is necessary.

To clarify the impact of mineral-related variables on their reduction extents and rates, redundancy analysis (RDA) was applied to the data collected from mediated electrochemical and microbial reduction experiments (SI, Section S4, Tables S4 and S5). To complement RDA, the significance of correlation was also analyzed through Pearson correlation analysis.^63^

**Figure 3:**
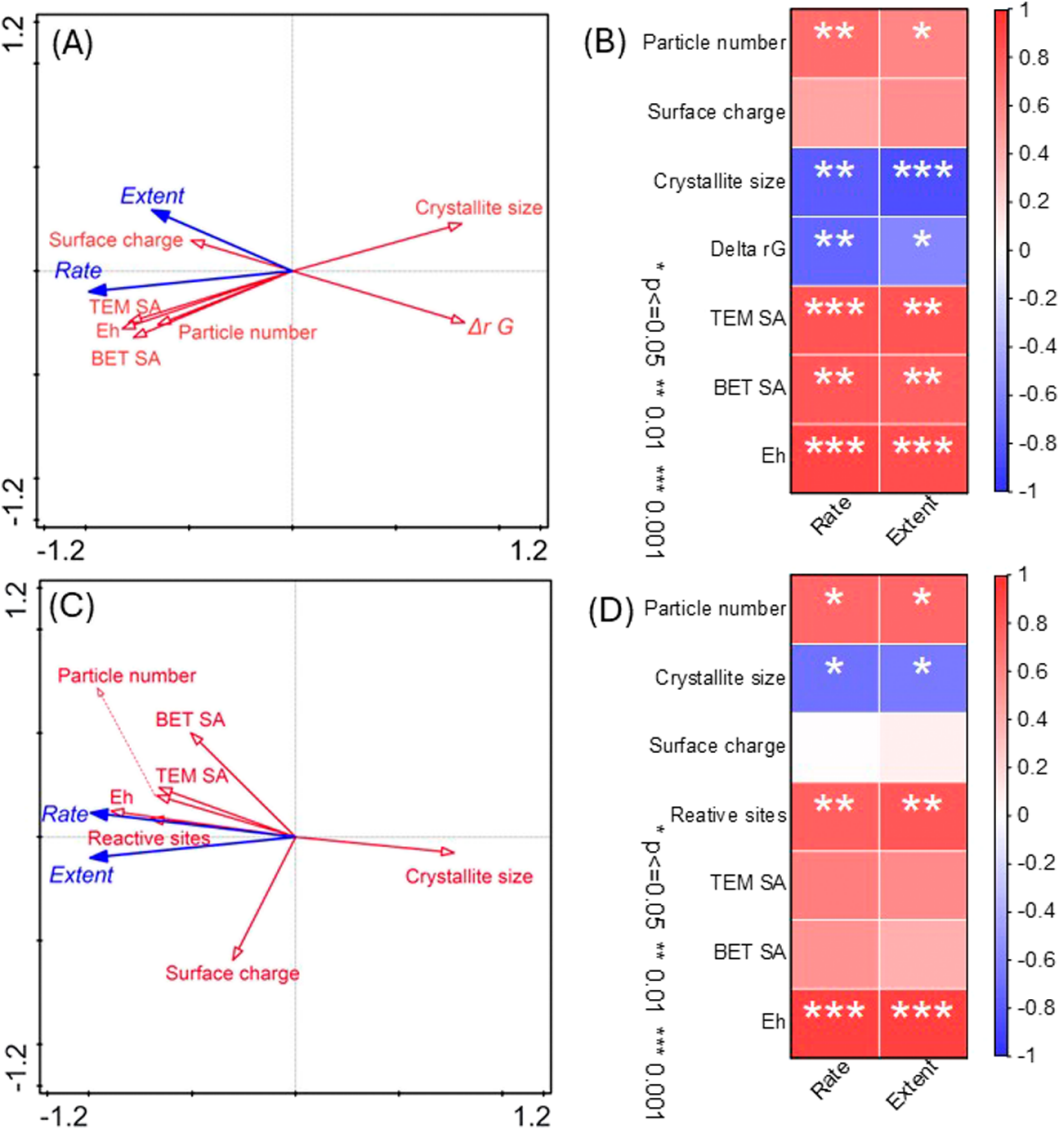
Results of redundancy analysis (A, C) and Pearson correlation analysis (B, D) for related variables and corresponding reduction extents and rate constants in the mediated electrochemical reduction (A, B) and microbial reduction (C, D). Blue solid lines with solid arrowheads in (A, C) represent reduction extents and rate constants as response variables. Red solid lines with unfilled arrowheads represent different interrelated explanatory variables. Stars in (B, D) represent significant correlation (*P* < 0.05; 0.01; 0.001), and the value beside the column represents the extent of correlation.

For mediated electrochemical reduction, the greatest impacts on rate and extent reflected by RDA were Δ_*r*_*G*, *E*_H_, TEM surface area, BET surface area, and crystallite size (Figure 3A). The acute angles (<90°) between red arrows for *E*_H_, BET surface area, TEM surface area, surface charge, and particle number with rate and extent reflected their positive correlations, and the obtuse angles (>90°) between red arrows for Δ_*r*_*G* and crystallite size with rate and extent indicated their negative correlations. The significance of correlations between the above interrelated variables and rate and extent is shown in Figure 3B (*P* < 0.05, 0.01, 0.005). The six variables mentioned above (particle number, crystallite size, Δ_*r*_*G*, TEM SA, BET SA, and *E*_H_) were all significantly correlated to both reduction rate and extent. In comparison, for microbial reduction experiments, the greatest impacts were *E*_H_, crystallite size, reactive sites, and BET SA and TEM SA (Figure 3C). Significant correlations were only found between rate and extent and particle number, crystallite size, reactive sites, aggregation size, and *E*_H_ (Figure 3D).

Collectively, the results from RDA and Pearson correlation analysis indicated that the thermodynamic driving force (represented by Δ_*r*_*G* and/or *E*_H_) and surface processes (represented by TEM surface areas (for MER) and reactive sites (for microbial Fe(III) reduction)) are the main controlling factors in reduction. This goes beyond the current understanding as past work has shown either (a) BET-based surface area is the only controlling factor^18,20^ or (b) when comparing different bulk minerals that thermodynamic driving force is the most important.^28,29^ Here, we show that thermodynamics plays a primary role, while surface area plays a secondary role, which can be applied to varying particle size and different mineral types.

Our results are supported by previous studies that highlighted thermodynamics and surface availability in controlling Fe(III) reduction. From a theoretical thermodynamics viewpoint, small particles are expected to have higher free energy and reduction potential than their large counterparts,^54,55^ thus making them more reactive and allowing for faster rates and higher extent of Fe(III) oxide reduction. This hypothesis was supported by the relationship between the reduction behavior and our calculated *E*_H_ and Δ_*r*_*G* values.

Additionally, surface area has been identified as a key controlling factor for both biotic and abiotic Fe(III) reduction in several papers.^18,20^ Our MER results also showed a positive correlation between surface area and Fe(III) reduction rates. Interestingly, in our microbial Fe(III) reduction experiments, it was the reactive site rather than the BET or TEM surface area that contributed more to the reduction rate and extent. Past studies^24,64^ have suggested that nanoparticles are prone to aggregation, strongly affecting their reactive surface area and further reactivity. Our batch MER experiments were carried out at a low initial Fe(III) concentration and with continuous stirring. Therefore, it is possible that goethite and hematite particles underwent slight aggregation, and thus, the reactive surface area may still be reasonably represented by the TEM and BET surface area measurements. Conversely, the significantly higher initial Fe(III) concentration (70× higher relative to MER experiments) and the stationary conditions maintained throughout the experiments can promote extensive nanoparticle aggregation. Further aggregation can be promoted by the presence of microbial cells and potential extracellular polymeric substances (EPS), which was supported by the tight association between cells and small particle aggregates (Gt_90 and Hm_8) and relatively loose association with large particle aggregates (Gt_2000 and Hm_40) determined by fluorescence microscopy (Figure S14). This aggregation may decrease the reactive sites on the nanoparticle surfaces, thereby limiting the accessibility of AQDS to these sites. Herein, the adsorption of Fe(II) by particles was measured and used as a proxy for reactive sites, so that the effect of aggregation on reactive sites could be accounted for. The RDA results demonstrated reactive sites correlated better with microbial Fe(III) reduction, compared to surface area.

When applying these results to the natural environment, note that environmental factors such as temperature, pH, and microbial community may affect reduction rate constants.^11,12,65^ For instance, relatively lower pH may promote reduction due to the availability of protons and more exposed reactive sites.^66^

### Microbial Fe(III) Reduction in Mixed Mineral Systems

**Figure 4:**
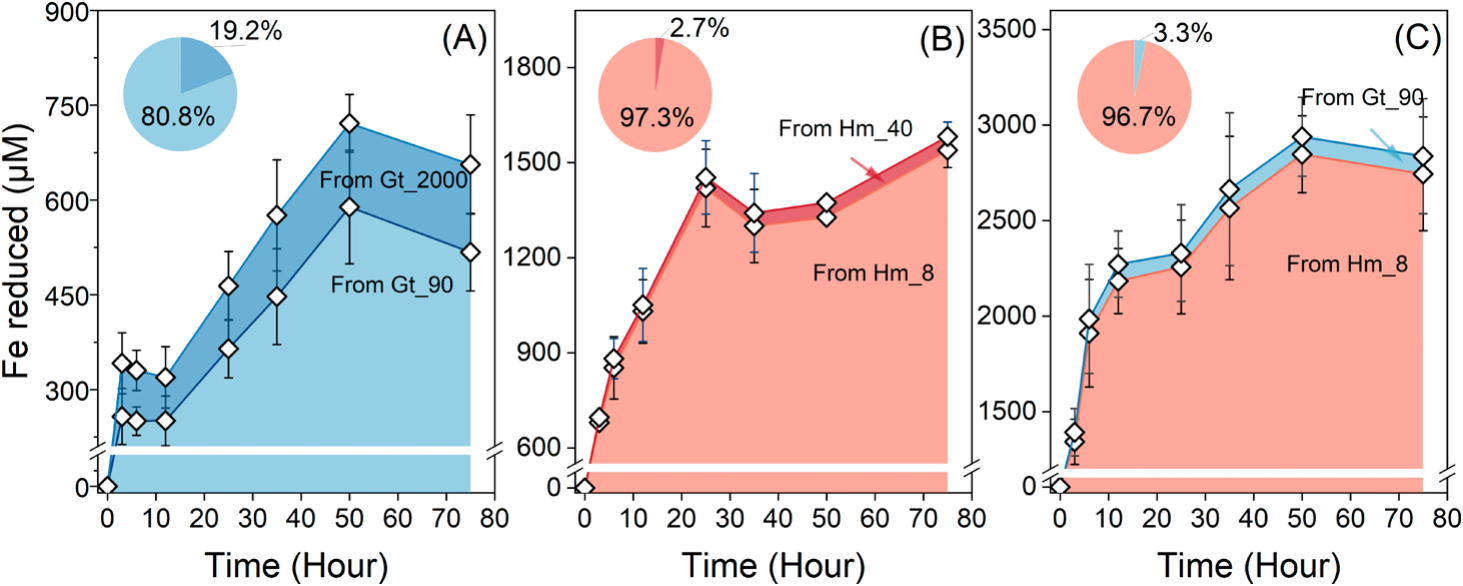
Reduced iron (Fe(II)) production was based on the isotopic composition of the microbially reduced Fe from reactors containing an isotopically labeled mix of different sizes of goethite and hematite. Data points correspond to an average of biological triplicates, and the error bars indicate standard deviations. Pie chart inside each panel illustrates fractions of Fe(II) derived from specific sized minerals at the final time point. Experimental conditions: 5 × 10^8^ cells mL^–1^*S. oneidensis* MR-1; 20 μM AQDS; pH 7; (A) 5 mM ^56^Gt_2000 and 5 mM ^NA^Gt_90; (B) 3.5 mM ^56^Hm_40 and 6.5 mM ^NA^Hm_8; (C) 4.5 mM ^NA^Gt_90 and 5.5 mM ^56^Hm_8.

An experimental system with iron (oxyhydr)oxides of different isotopic compositions allowed us to track the origin of the Fe reduction during incubation with microorganisms. In mixed mineral systems, we first performed linear combination between *E*_H,__mix_ and SA_mix_ and overall rate constants, which suggest no clear tendency compared to single mineral systems (SI, Figure S15). To determine whether smaller particles of goethite and hematite are preferentially reduced by *S. oneidensis* MR-1 in mixed mineral systems, we calculated the fraction of Fe reduced from specific sized minerals based on the ^54^Fe concentration of the total Fe(II) produced (details in SI, Section S5). We observed that although both minerals in each mixed mineral system were reduced simultaneously, the majority of the Fe(II) produced, ranging from 81 to 97%, was attributed to the reduction of smaller minerals (Figure 4). We further determined both reduction extents and rate constants for each mineral to compare between single and mixed mineral systems (SI). Table S6 shows that the reduction extents of small minerals are always higher than those of large minerals, which is overall consistent with the previous single mineral systems (Tables 1 and S2). In addition, the reduction extents of small minerals in mixed mineral systems are slightly higher than their reduction extent in single mineral systems under the same environmental conditions. For instance, Hm_8 in Hm/Gt mixed systems is reduced 2× more compared to single mineral systems. In contrast, the reduction extents of large minerals are much lower than those in single mineral systems. Specifically, Gt_2000, Hm_40 and Gt_90 in mixed mineral systems are reduced 3 × , 6× and 5× less than in single mineral systems. Figure S16 shows the fraction of Fe(II) derived from each mineral in mixed mineral systems, demonstrating that 81, 97, and 97% of Fe(II) can be attributed to reduction of smaller minerals. These findings confirmed that smaller minerals are preferentially reduced by *S. oneidensis* MR-1 in mixed mineral systems and even to a higher extent than that solely predicted based on single mineral systems.

The different reduction behaviors can also be observed in the reduction rate constants (SI, Table S6). When considering single mineral systems, reduction rate constants of Gt_90, Hm_8, and Hm_8 were 1.5×, 3.6×, and 3.2× greater than those of Gt_2000, Hm_40, and Gt_90, respectively. When reduction happened in mixed mineral systems, reduction rate constants of Gt_90, Hm_8, and Hm_8 were 6×, 21×, and 23× greater than their large-sized counterparts. This significant difference was attributed mostly to a pronounced reduction suppression of large particles and secondarily to a slight promotion of reduction of small particles.

### Possible Mechanisms for Preferential Microbial Reduction of Small-Sized Particles over Large Particles in Mixed Mineral Systems

Based on our RDA and Pearson correlation analysis above, *E*_H_ and reactive sites were the most important variables for microbial reduction in single mineral systems. Thus, they also likely contribute to differences in the reduction extent and rate in our mixed mineral systems. In our mixed mineral systems, the reduction of small particles is expected to be more thermodynamically favorable (higher *E*_H_ values). In addition, given the initially added concentration and surface area of specific iron (oxyhydr)oxides in mixed systems, small particles had 2× to 7× higher reactive sites compared to large particles, which can contribute to differential reduction.

The potential preferential adsorption of AQDS between particles may also contribute to differential reduction of the Fe(III) mineral. Negatively charged AQDS acts as an electron shuttle between Fe(III)-reducing bacteria and minerals.^66,67^ The point of zero charge of our synthesized minerals were 9.2 and 8.5 for Gt_2000 and Gt_90, and 9.2 and 8.0 for Hm_40 and Hm_8, respectively (SI, Figure S17). It has been extensively shown that negatively charged organic molecules adsorbs on iron (oxyhydr)oxide via electrostatic interactions.^68,69^ Thus, small particles which exhibit more positively charged surfaces than their large counterparts at our experimental pH (7.0) may provide higher electrostatic attraction for AQDS. In addition, the number of reactive surface sites (>10^18^) on small particles is an order of magnitude higher than that of added AQDS molecules (10^17^) (SI, Table S5). If each reactive site can bind only one AQDS molecule, then there will be insufficient AQDS to react with large particles. Consequently, this could inhibit the reduction of large particles in mixed mineral systems.

The presence of defects may also lead to a preferential reduction. It has been found that defects can facilitate electron transfer in both abiotic and biotic systems.^38,70^ In our study, the presence of defects in small particles was confirmed through XRD and Mössbauer spectroscopy data. These defects likely contribute to the increased electron transfer in smaller particles, making them more prone to reduction.

### Possible Mechanisms for Suppressed Microbial Reduction of Large-Sized Particles in Mixed Mineral Systems Compared to Single Mineral Systems

Interestingly, the observed pronounced suppression of reduction in large-sized particles occurs not only in comparison to small particles but also when compared to particles of the same size within homogeneous single mineral systems. In a system with a broad particle size distribution, such as the mixed mineral system in this study, Ostwald ripening is a process worth considering. As far as we could measure, Ostwald ripening did not play a major role. We found no clear evidence that large particles in mixed mineral systems became noticeably larger (Figure S18), when compared to those in single mineral systems. This is supported by our earlier findings^71,72^ that the length of the particles of nanoparticulate goethite does not change substantially, even in the presence of Fe^2+^. Below, we propose three explanations for suppressed reduction of large particles (SI, Figure S20).

Herein, we speculate that preferential reduction can be ascribed to varied aggregation patterns in mixed mineral systems. Mineral mixtures can result in heteroaggregation due to their morphological variations and surface charge disequilibrium.^41,73,74^ In our present study, the mixed minerals displayed shifts toward larger aggregate sizes relative to the single minerals (Detailed data and description in SI, Section S6). This could result in increased physical coverage of reactive sites and a decrease in the aggregate pore throat, which slows diffusion of shuttle molecules such as AQDS (0.5–1.5 nm size) (SI, Section S6). However, it must be noted that DLS data can indicate only the hydrodynamic diameter of an aggregate in the absence of microbes, without any knowledge on the aggregate morphology and compaction factor (i.e., loose versus compact packing) as well as its association with microbial cells. Further exploration using cryogenic transmission electron microscopy (cryo-TEM) and small-angle X-ray scattering (SAXS) is needed to test this hypothesis.

The second speculation is related to the electron transfer (ET) process, leading to the impeded reduction of large particles and the enhanced reduction of small particles. Previous studies have substantiated the existence of electron transfer via electron hopping between different microbial species,^75−78^ between minerals and minerals,^79,80^ as well as within iron (oxyhydr)oxides.^81^ More recently, it has been revealed that directional long-distance ET can occur along a redox gradient, driven by differences in reduction potentials, with electrons moving from lower to higher reduction potentials.^82^ Therefore, large particles with lower reduction potentials can facilitate directional electron transfer toward small ones, when these minerals are present as heteroaggregates.

The third speculation is attributed to the disproportionate adsorption capacity and affinity toward dissolved Fe(II) among different sized minerals. It has been reported that dissolved Fe(II) adsorbs onto residual minerals, thereby inhibiting their further reduction.^19,83^ As discussed above, large particles were less positively charged than small ones, as indicated by the zeta potentials of 29.8 mV for Hm_40 and 34.5 mV for Hm_8 (SI, Table S5). The less positive surface charge can lead to released Fe^2+^ being more easily attracted to their surface. This selective attraction may block reactive surface sites, thereby forming a passivated layer that inhibits further reduction. This passivation effect may result in slower and less extensive reduction of large particles in mixed mineral systems, compared to single mineral systems.

## Environmental Implications

The reduction of iron (oxyhydr)oxides is one of the most important biogeochemical processes on Earth that significantly impacts the fate and bioavailability of nutrients, trace elements and pollutants.^7,12^ Iron(III) mineral reduction was originally thought to be predominantly influenced by mineral phases and crystallinity. Poorly crystalline minerals, like ferrihydrite, were found to be much more susceptible to reduction compared to highly crystalline minerals, such as lepidocrocite, magnetite, goethite and hematite.^14,21,24,84,85^ Our results show that particle size plays an important role in controlling reduction kinetics, potentially outweighing other factors such as crystallinity and the mineral phase. For instance, hematite (8 nm) in our studies was more susceptible to reduction compared to goethite of larger sizes (30, 90, and 2000 nm), regardless of electrochemical and microbial reduction. In addition, in heterogeneous environments where minerals of varying sizes and phases are likely to be present together, our results suggested an enhancement of the reduction of small particles and a strong suppression of the reduction of larger particles.

Our findings have significant implications for the fate and behavior of nutrients, pollutants, and trace elements associated with iron (oxyhydr)oxide minerals. Iron (oxyhydr)oxides have been widely employed in environmental remediation and agricultural production to remove contaminants and enhance soil fertility, due to their high sorption capacities.^86−88^ However, nanoparticles are more susceptible to undergoing extensive reduction under anoxic conditions, affecting the mobility and bioavailability of trace elements and pollutants in soils and aquatic systems. These interactions suggest that when accounting for particle size of iron minerals, more accurate predictions of trace element behavior under redox fluctuating conditions can be made. In addition, the higher reactivity of smaller particles should be incorporated into reactive transport models to better estimate the flux of elements across the redox boundaries in natural systems.

Environmental studies focused on particles suspended in aquatic bodies commonly utilize 0.45 or 0.22 μm membrane filters to separate solid and dissolved aqueous phases, which fails to account for nanoparticles that can pass through filters. To illustrate the impact of particle size on the reducible phase, we developed a model assuming a total Fe concentration of 10 mM, with particle sizes distributed across three ranges: 1–10, 10–100, and >100 nm. By applying reduction extents from our experiments, we approximated the reducible fraction for each size range, allowing us to calculate the contributions of different particle sizes to the overall reducible Fe phase (details in SI, Section S7). The model results clearly demonstrate that neglecting the size effect and not considering particles <0.45 μm can lead to a significant underestimation (7× for hematite and 14× for goethite) of the reducible solid phase in natural aquatic systems (Figure S21). This highlights the need for more advanced techniques, such as field-flow fractionation (FFF) and single particle inductively coupled plasma mass spectrometry (SP-ICP-MS), which can more accurately detect and characterize nanoparticles. Furthermore, it is important to recognize that naturally formed iron (oxyhudr)oxides may exhibit different behaviors than synthesized iron oxides under laboratory conditions. Therefore, future field studies are required to validate the behavior of natural iron (oxyhydr)oxides under complex environmental conditions.

## Supporting Information Available

Details about synthesis and characterization of nanoparticulate goethite and hematite, mediated electrochemical reduction of iron oxides, data collection and calculation for RDA and Pearson correlation analysis, and calculation of the origin of reduced Fe based on their isotope concentration (PDF)
