# Supplementary material for: Size-Dependent Reduction Kinetics of Iron Oxides in Single and Mixed Mineral Systems
Source: Environ Sci Technol. 2025 Jan 29;59(5):2519–30. doi: 10.1021/acs.est.4c08032 (PMC11823449; doi:10.1021/acs.est.4c08032)
Supplement: Supplementary file 1 — es4c08032_si_001.pdf [file es4c08032_si_001.pdf]

Supporting Information for

Environmental Science & Technology

Size-dependent Reduction Kinetics of Iron (Oxyhydr)oxides in Single and Mixed Mineral Systems

Xiyang Xu†, Muammar Mansor†, Guoxiang Li‡, Tsz Ho Chiu†, Stefan B. Haderlein‡, Andreas Kappler†, §, Prachi Joshi*, †

† Geomicrobiology, Department of Geosciences, University of Tübingen, 72076 Tübingen, Germany

‡ Environmental Chemistry and Mineralogy, Department of Geosciences, University of Tübingen, 72076 Tübingen, Germany

§ Cluster of Excellence: EXC 2124: Controlling Microbes to Fight Infection, Tübingen, Germany

* Corresponding author: Prachi Joshi; E-mail: Prachi.joshi@uni-tuebingen.de

The supporting information contains: 32 pages, 14 figures, and 5 tables.

Content

S1 Synthesis and characterization of goethite and hematite S3

S1.1 Synthesis S3

S1.2 Characterization S4

S2 Mediated electrochemical reduction of iron (oxyhydr)oxides..............................S12

S3 Microbial Fe(III) reduction of iron (oxyhydr)oxides...S17

S4 Data collection and calculation for RDA and Pearson correlation analysis S19

S5 Calculation of the origin of reduced Fe based on their isotope concentration S27

S6 Possible mechanisms for preferential microbial reduction..................................S32

S7 Environmental implications S36

References S38

S1 Synthesis and characterization of goethite and hematite

S1.1 Synthesis

Natural Fe abundance (NA) and ^{56}Fe enriched goethite and hematite with varying particle sizes were synthesized following published methods[1-3]. The detailed steps for ^{NA}Fe synthesized Gt_2000, Gt_90, Gt_30, Hm_300, Hm_40, and Hm_8 are described below. ^{56}Fe-enriched Fe minerals were prepared in the same way, but using enriched ^{56}Fe instead of ^{NA}Fe solutions.

Gt_2000 was synthesized by rapidly adding 180 mL of 5 M NaOH into 100 mL of 1 M $Fe(NO_3)_3 \cdot 9H_2O$ solution. The mixture was diluted with distilled water to a final volume of 2 L and was held in a closed polyethylene flask at 70°C for 70 hours.

Gt_90 was synthesized by adding 100 mL of 0.48 M $NaHCO_3$ dropwise into a continuously stirred solution of 100 mL of 0.40 M $Fe(NO_3)_3 \cdot 9H_2O$. The suspension was collected into 250 mL Nalgene bottles and microwaved in 20 s intervals until boiling occurred with venting and shaking between each interval. Immediately after boiling, the suspension was plunged into an ice bath until it reached 20°C. The cooled suspension was placed into a dialysis bag (MWCO =14 kD) for three days to remove the counter ions. The water was changed three times per day. After dialysis, the pH of the suspension was quickly adjusted to 12 using 5 M NaOH. The suspension was heated at 90°C for 24 hours, followed by centrifugation and washing to remove residual ions.

Gt_30 was synthesized by dissolving 283 g of $Fe(NO_3)_3 \cdot 9H_2O$ in 350 mL of 2 M HNO_3, followed by dilution with distilled water to a total volume of 1.4 L. This solution was

mixed with an equivalent volume of 1 M NaOH under vigorous stirring. The pH was adjusted to 1.7-1.8, and the suspension was maintained at room temperature for 50 days. Afterwards, the supernatant was discarded, and the precipitates were collected by centrifugation and subsequently washed with deionized (DI) water.

Hm_300 was synthesized by first heating 250 mL of 0.002 M HCl to 98°C, followed by the addition of 2.02 g of $Fe(NO_3)_3 \cdot 9H_2O$ with vigorous shaking of the flask. The resulting solution was held in a closed vessel at 98°C for 10 days.

Hm_40 was synthesized by heating 200 mL of 0.2 M $Fe(ClO_4)_3 \cdot 9H_2O$ solution at 98°C for 7 days.

Hm_8 was synthesized by slowly dripping 60 mL of 1 M $Fe(NO_3)_3 \cdot 9H_2O$ into 750 mL boiling DI water with continuous stirring. The suspension was removed from heat after drip solution was consumed and was cooled down to room temperature. The cooled suspension was placed into a dialysis bag (MWCO =14 kD) for five days, followed by the collection of nanoparticles via ultracentrifugation.

S1.2 Characterization

The purity of the synthesized iron (oxyhydr)oxides was determined using micro X-ray diffraction and ^{57}Fe Mössbauer spectroscopy. The morphologies and particle size distributions of the iron (oxyhydr)oxides were characterized using scanning electron microscopy (SEM) and transmission electron microscopy (TEM). The measured BET surface area was determined by N_2 sorption at 77 K. The zeta potentials and aggregate

size of the iron (oxyhydr)oxides were measured by dynamic light scattering (Malvern Zetasizer Nano ZS).

Micro X-ray diffraction (μ-XRD): Dry materials were analyzed on a Bruker's D8 Discover GADDS XRD2 micro-diffractometer equipped with a standard sealed tube with a Co-anode at parameters of 30 kV/30mA. The total time measurement was 240 sec at two detector positions, 15° and 40°. XRD patterns are shown in Fig. S1.

Mössbauer spectroscopy: Dried sample powder was loaded into 1 cm^2 Plexiglas holders. Spectra were collected at 77K using a constant acceleration drive system (WissEL) in the transmission mode with a $^{57}Co/Rh$ source. Analysis was carried out using a Recoil (University of Ottawa) and the Voigt Based Fitting (VBF) routine[4]. The Lorentzian linewidth was fixed at 0.123 mm/s during fitting, as this value was the linewidth measured on the spectrometer using an ^{57}Fe foil with an ideal thickness. Mössbauer spectra are shown in Fig. S2 with parameters presented in Table S1.

Microscopy: For SEM analysis, samples were placed onto an aluminum stub using carbon adhesive tape, followed by a depositional coating of gold. The samples were then investigated using a Zeiss Crossbeam 550L Focused Ion Beam scanning electron microscope (SEM) (Zeiss, Germany) with an acceleration voltage of 2.0 kV. For TEM analysis, samples were prepared on a carbon TEM grid and investigated using transmission electron microscopy (JEOL, Japan) with an acceleration voltage of 120 kV. Representative images of our iron (oxyhydr)oxide samples in single and mixed mineral systems are shown in Fig. S3 and Fig. S4, respectively.

Size distribution: Primary particle size distributions were analyzed via SEM and TEM images using Nano Measurer (1.2). Since goethite particles are rod-shaped, we thus characterized their size using length, width and aspect ratio. For cubic hematite particles, we characterized their size using diameter. Size distribution based on SEM/TEM images is shown in Fig. S5.

Dynamic light scattering (DLS) and Zeta potential (ZP): DLS and ZP measurements were performed using a Malvern Zetasizer Nano ZS (Malvern Instruments), to investigate the (hydrodynamic) size and surface charge of the nanoparticulate iron (oxyhydr)oxides, respectively. Specifically, the minerals suspensions for DLS and ZP analysis consisted of iron (oxyhydr)oxides (5 mM), HEPES buffer (15 mM, pH=7), and NaCl (10 mM). All suspensions were prepared 24 hours before DLS analysis and were sonicated for 15 minutes to disperse aggregates right before the measurements. For DLS measurements, the suspension was vortexed for 30 seconds followed by 20 seconds non-shaking to allow any extremely large aggregates to settle out of the suspension. Afterwards, a well-mixed 1 mL aliquot of the suspension was loaded into a disposable cuvette and placed into the Zetasizer. The Z-average size is derived from a cumulant analysis of the measured correlation curve. Intensity-weighted size distributions were obtained by the regularized non-negatively constrained least-squares (NNLS) method. For ZP measurements, the pH of the suspensions (ranging from 4 to 12) was manually adjusted using HCl and NaOH (10 mM and 100 mM) and measured using a pH meter. Then, a 1 mL aliquot of the suspension was transferred into disposable folded capillary

cells and placed into the Zetasizer. The zeta potential was obtained from the electrophoretic mobility by the Smoluchowski equation[5].

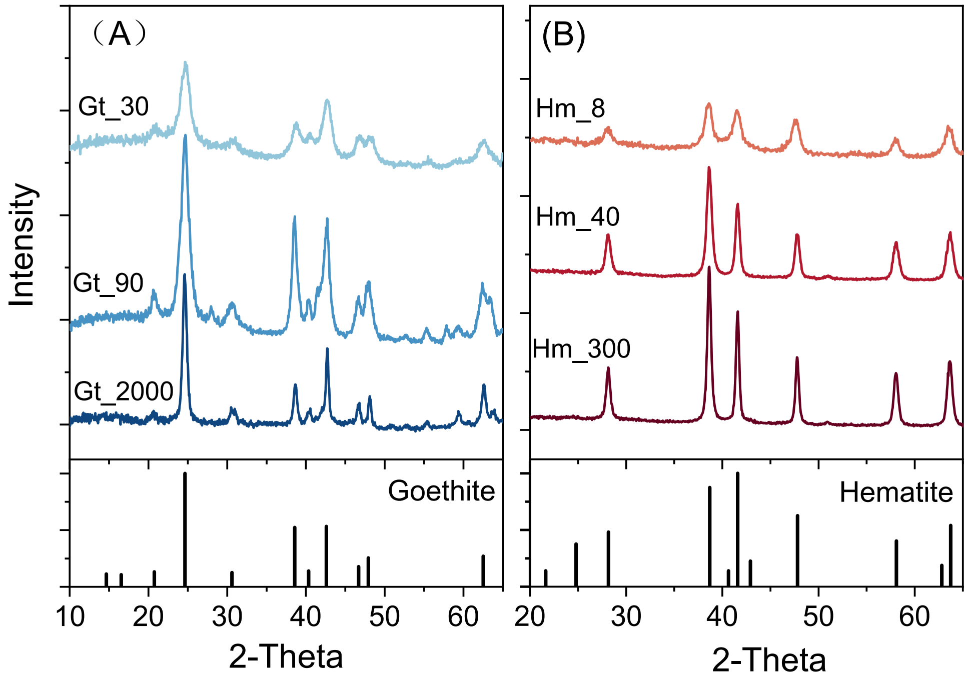

Figure S1. XRD patterns of the synthesized goethite (A) and hematite (B) as a function of particle size. The XRD reflections widen with decreasing particle sizes.

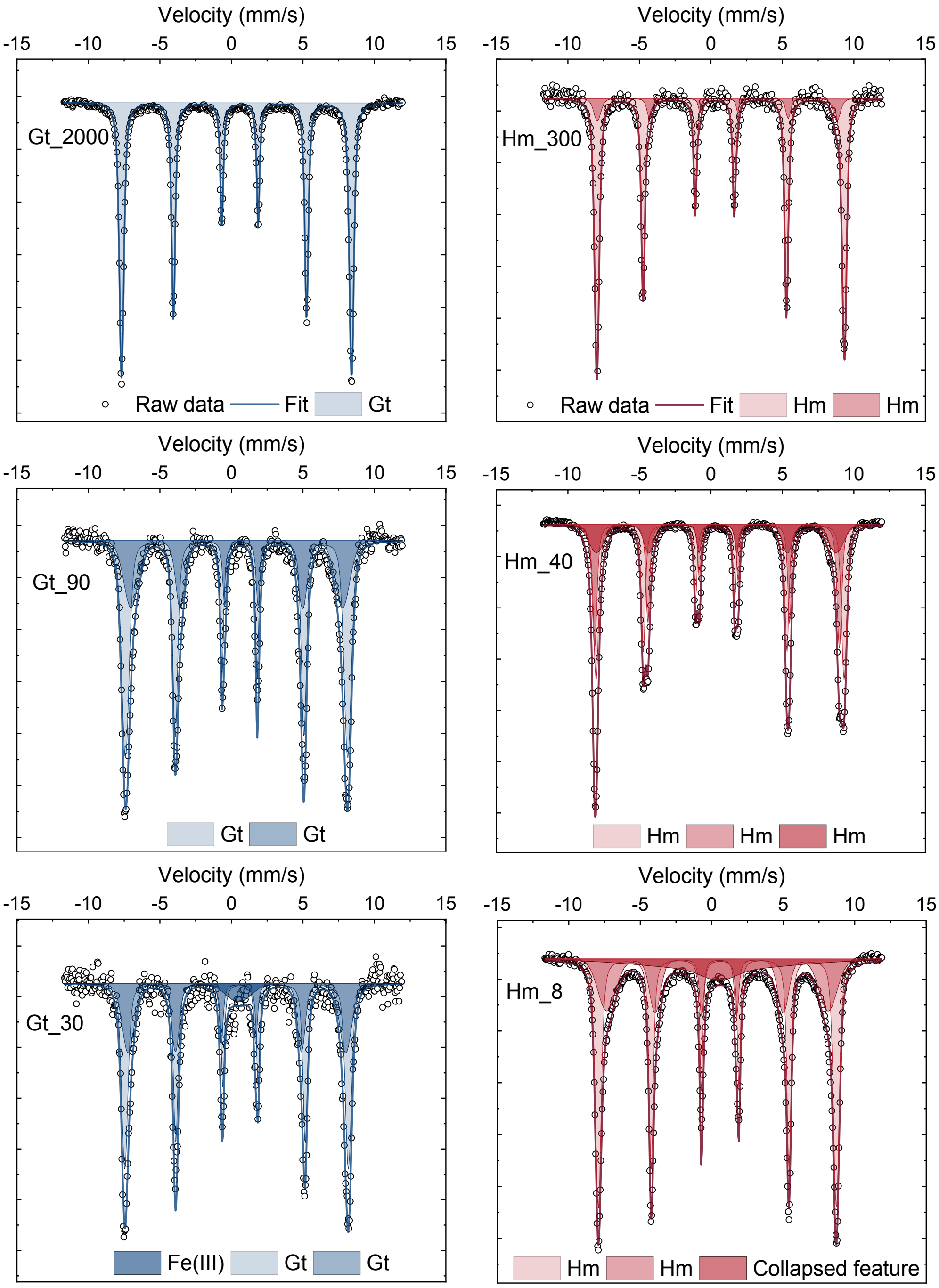

Figure S2. Mössbauer spectra of the synthesized goethite (A) and hematite (B) at 77 K. Symbols represent experimental raw data, fit curves represent the total fit, and the shaded sextet represents the hyperfine field distribution (HFD).

Table S1. Mössbauer parameters for the synthesized goethite and hematite at 77 K.

Sample	CS [a]	QS [b]	H [c]	Aera
Gt_2000	0.48	-0.12	49.9	100.0
Gt_90	0.49	-0.14	49.3	62.5
	0.43	-0.06	48.8	37.5
Gt_30	0.5	0.47		5.1
	0.49	-0.14	48.5	57.3
	0.44	-0.06	47.2	37.6
Hm_300	0.48	0.20	53.7	88.8
	0.49	-0.06	51.9	11.2
Hm_40	0.47	0.17	53.8	45.1
	0.49	-0.06	53.1	33.7
	0.43	-0.06	52.0	21.1
Hm_8	0.49	-0.10	51.6	53.7
	0.46	-0.06	48.1	30.2
	0.5	0.00	0	16.1

[a] Center shift [mm/s]; [b] Quadrupole splitting [mm/s]; [c] Hyperfine field [T];

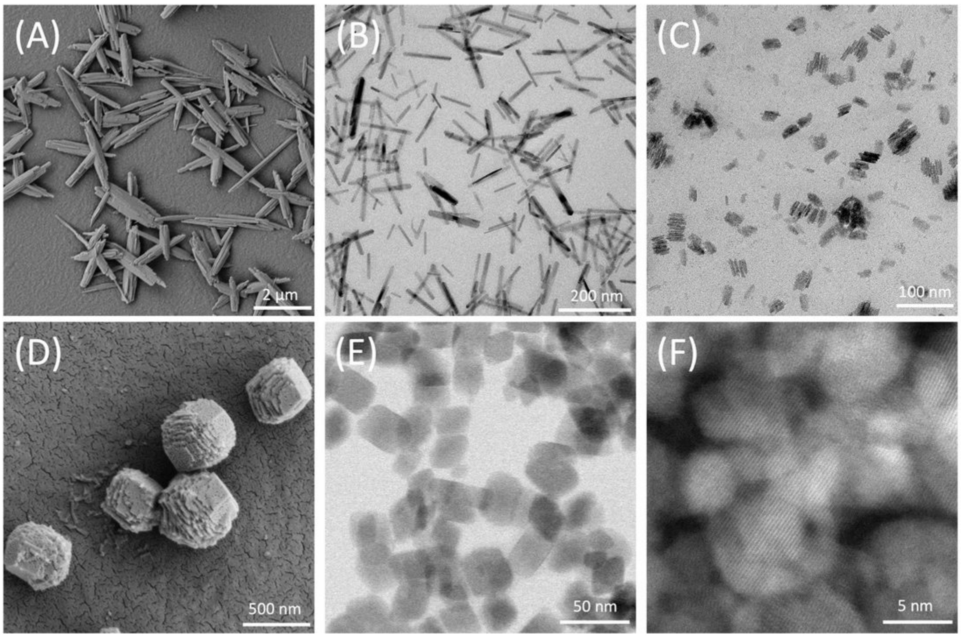

Figure S3. Representative SEM and TEM images of synthesized Gt_2000 (A), Gt_90 (B), Gt_30 (C), Hm_300 (D), Hm_40 (E), and Hm_5 (F) nanoparticles. Note the different scale bars in the images.

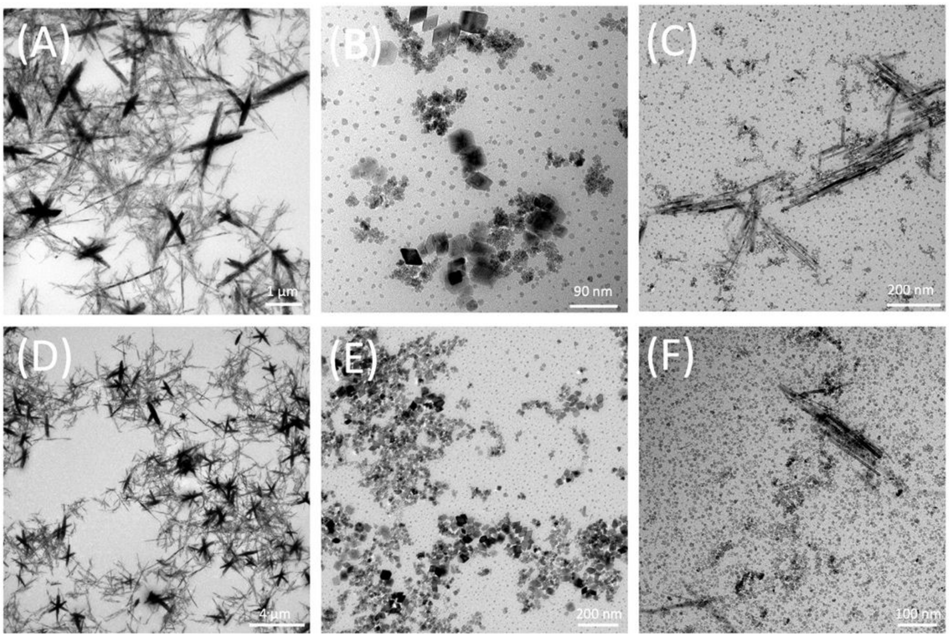

Figure S4. Representative TEM images of mixed mineral systems Gt_2000 & Gt_90 (A, D), Hm_40 & Hm_8 mixture (B, E), and Gt_90 & Hm_8 (C, F). Please note the differences in the scale bar for the same mixture.

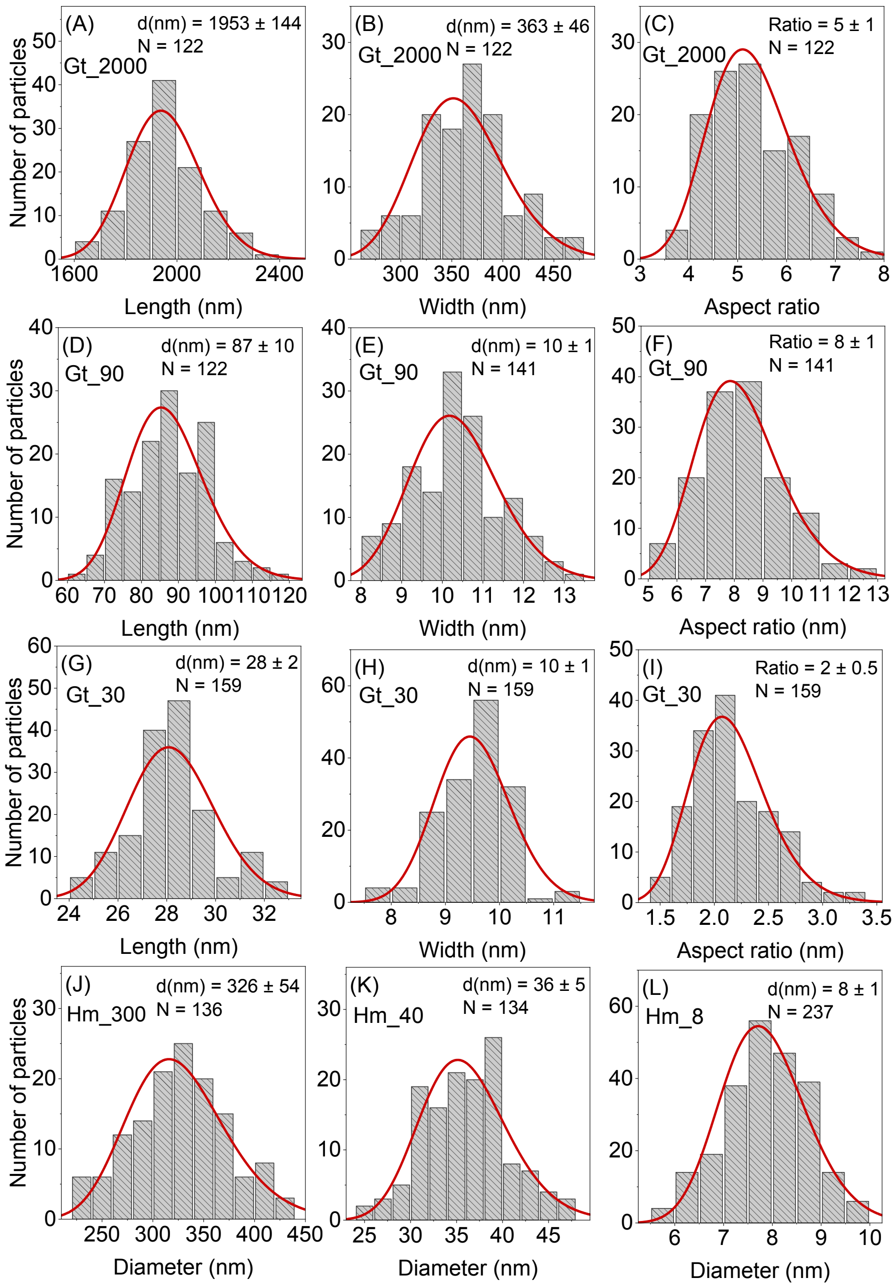

Figure S5. Particle size distribution of goethite and hematite obtained from TEM or SEM images. Length, width and aspect ratio of Gt_2000 (A, B, C), length, width and aspect ratio of Gt_90 (D, E, F), length, width and aspect ratio of Gt_30 (G, H, I), diameter of Hm_300 (J), diameter of Hm_40 (K), diameter of Hm_8 (L). The parameters d(nm) represent the mean diameter while *N* represents the number of counted primary particles.

S2 Mediated electrochemical reduction of iron (oxyhydr)oxides

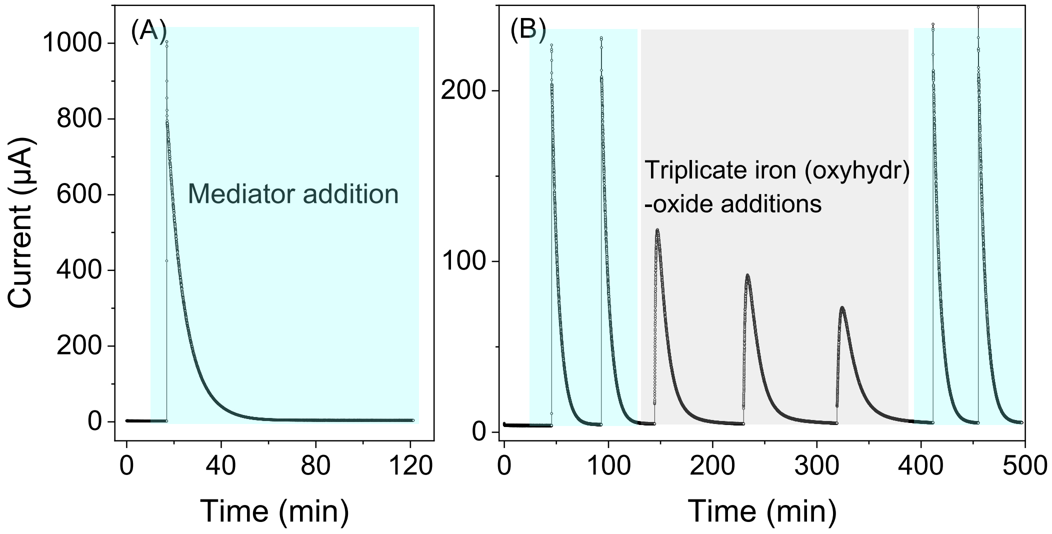

Figure S6. Addition scheme of mediator and iron (oxyhydr)oxides in MER experiment of Gt_2000 at pH 7.0 (E_H^{MER} = -0.53 V vs SHE) as an example. MER was conducted with an initial large addition (400 μL) of mediator (10 mM) (A); followed by second and third small addition (100 μL) of mediator, after which triplicate additions (100 μL) of iron (oxyhydr)oxides (10 mM) were made, and finally another duplicate small addition (100 μL) of the mediator (B) were performed to complete the process. Note the difference in the scalings of the y axes in panels.

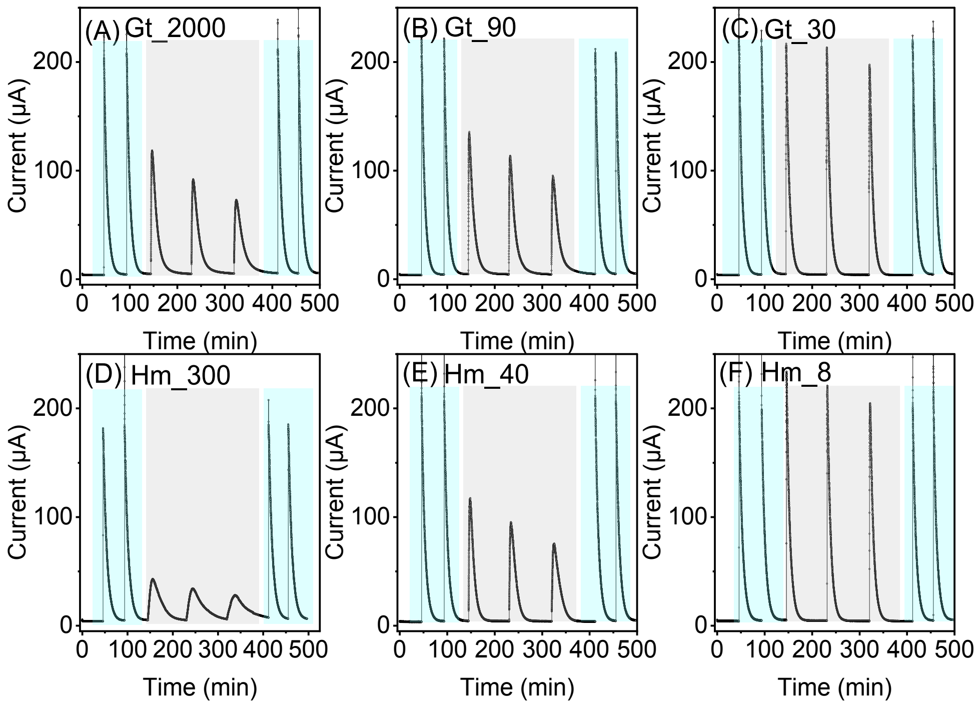

Figure S7. Current response to additions of mediator and iron (oxyhydr)oxides in mediated electrochemical reduction experiments at pH 7.0 (E_H^{MER} = -0.53 V vs SHE). The peaks in the blue-shaded regions represent the response to the mediator addition, and the peaks in the gray-shaded regions represent the response to the iron (oxyhydr)oxide addition. Gt_2000 (A), Gt_90 (B), Gt_30 (C), Hm_300 (D), Hm_40 (E), Hm_8 (F). For all three iron (oxyhydr)oxide additions, the peak heights decreased and the peak widths increased with increasing activities of Fe^{2+}, which accumulated from iron (oxyhydr)oxide reductive dissolution in the cells.

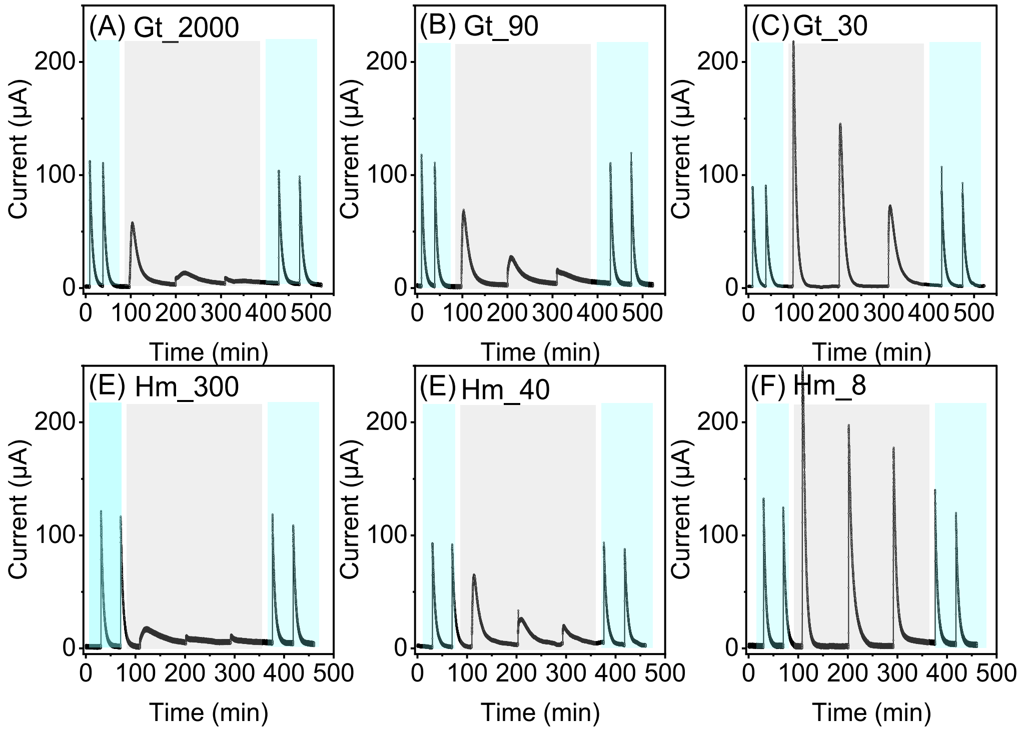

Figure S8. Current response to additions of mediator and iron (oxyhydr)oxides in mediated electrochemical reduction experiments at pH 7.0 (E_H^{MER} = -0.35 V). The peaks in the blue-shaded regions represent the response to the mediator addition, and the peaks in the gray-shaded regions represent the response to the iron (oxyhydr)oxide addition. Gt_2000 (A), Gt_90 (B), Gt_30 (C), Hm_300 (D), Hm_40 (E), Hm_8 (F).

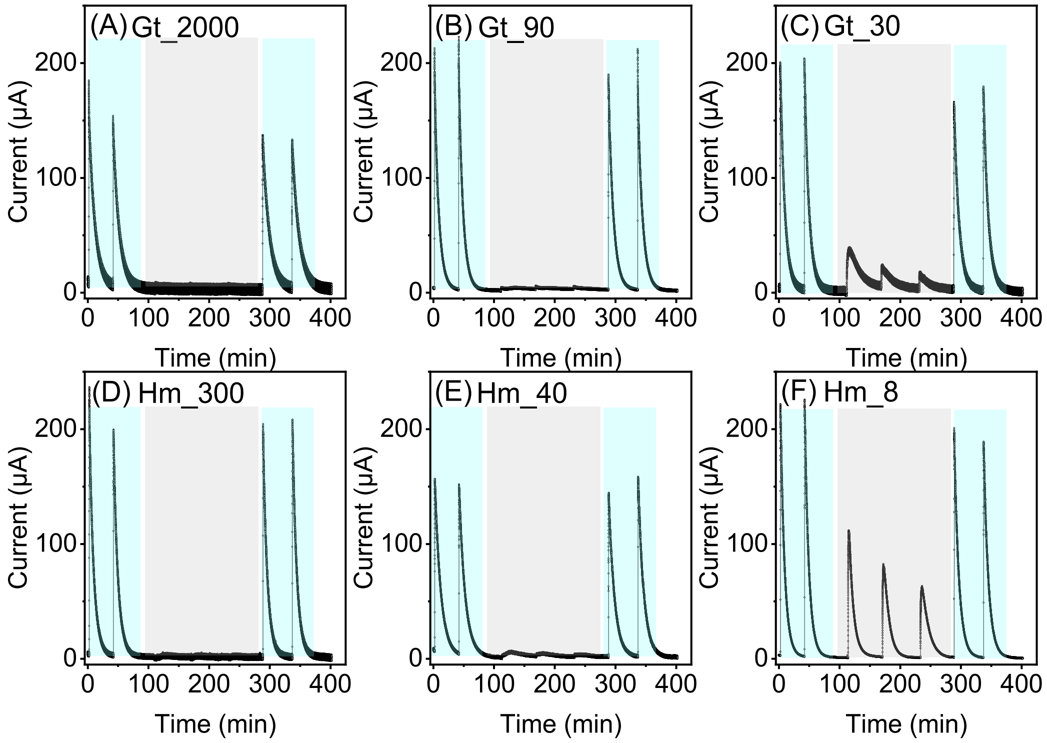

Figure S9. Current response to additions of mediator and iron (oxyhydr)oxides in mediated electrochemical reduction experiments at pH 7.0 (E_H^{MER} = -0.25 V). The peaks in the blue-shaded regions represent the response to the mediator addition, and the peaks in the gray-shaded regions represent the response to the iron (oxyhydr)oxide addition. Gt_2000 (A), Gt_90 (B), Gt_30 (C), Hm_300 (D), Hm_40 (E), Hm_8 (F).

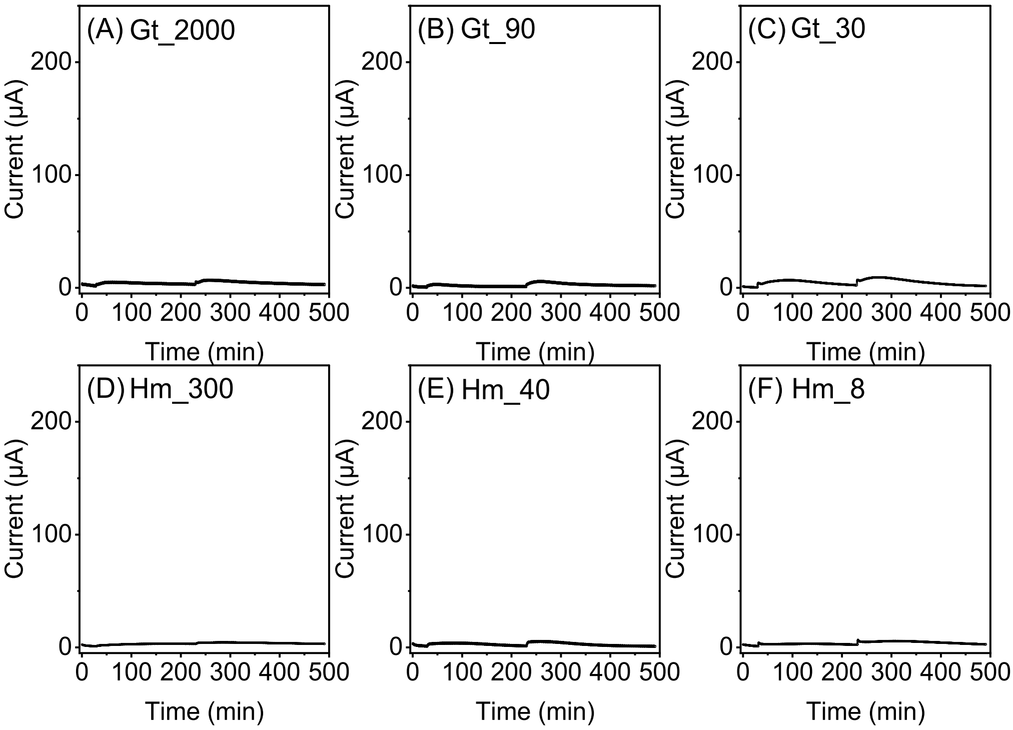

Figure S10. Current response to additions of iron (oxyhydr)oxides in electrochemical reduction experiments at pH 7.0 (E_H^{MER} = -0.35 V). Gt_2000 (A), Gt_90 (B), Gt_30 (C), Hm_300 (D), Hm_40 (E), Hm_8 (F). For all iron (oxyhydr)oxide additions, the short and broad peaks relative to the responses in cells with mediators (figures S7-S9) indicate that the reduction of iron (oxyhydr)oxides is far slower without mediators in the working cells.

Table S2. Reduction extents of goethite and hematite as a function of particle size

Minerals	Microbial reduction [a]	Mediated electrochemical reduction		
		E_H^{MER}= -0.25 V [b]	E_H^{MER}= -0.35 V [c]	E_H^{MER}= -0.53 V [d]
	%	%	%	%
Gt_2000	6.3 ± 0.3	0.0	61.7 ± 2.2	98.4
Gt_90	10.5 ± 0.5	4.1	67.7 ± 2.4	92.5
Gt_30	16.8 ± 0.7	45.3	100.2 ± 2.7	92.5
Hm_300	2.7 ± 0.3	0.00	35.2 ± 1.8	90.6
Hm_40	7.5 ± 0.4	13.8	62.3 ± 1.6	91.9
Hm_8	21.7 ± 1.2	54.9	98.1 ± 1.1	91.9

[a] Microbial reduction was carried out in triplicates. The values represent the mean ± standard deviation for a given mineral. [b] For all iron (oxyhydr)oxides (E_H^{MER} = -0.25 V), MER experiments were performed in individual cells. [c] For all iron (oxyhydr)oxides (E_H^{MER} = -0.35 V), MER experiments were performed in two separate electrochemical cells, reported reduction extents were the average of the duplicate measurements ± range. [d] For all iron (oxyhydr)oxides (E_H^{MER} = -0.53 V), MER experiments were performed in individual cells.

S3 Microbial Fe(III) reduction of iron (oxyhydr)oxides

To study extents and kinetics of microbial reduction of iron (oxyhydr)oxides, we performed cell suspension experiments using the fermentative Fe(III)-reducer *Shewanella oneidensis* MR-1. The cells were revived from a frozen stock culture maintained in the Geomicrobiology laboratory. A pre-culture was grown under oxic conditions using Luria-Bertani (LB) liquid medium. which contains 10 g/L peptone, 5 g/L yeast extract, and 10 g/L NaCl. The pre-culture was incubated for 24 hours at 28°C. Following incubation, cells were harvested by centrifugation at 4300 × g (10 mins) and washed twice using buffer solution to remove residual LB medium. The optical density (OD) of the cell suspension was measured at 600 nm, with an OD value of 1.5 corresponding to a cell concentration of 10^9 cells/mL. Next, the resulting cell pellets were resuspended in N_2-flushed, autoclaved buffer solution to achieve stock concentration. The resuspended cell solution was flushed with nitrogen gas (N_2) for 5 minutes, next to a Bunsen burner flame to minimize contamination. This stock cell suspension was temporarily stored (<1 hour) at room temperature before being used in batch microbial Fe(III) reduction experiments.

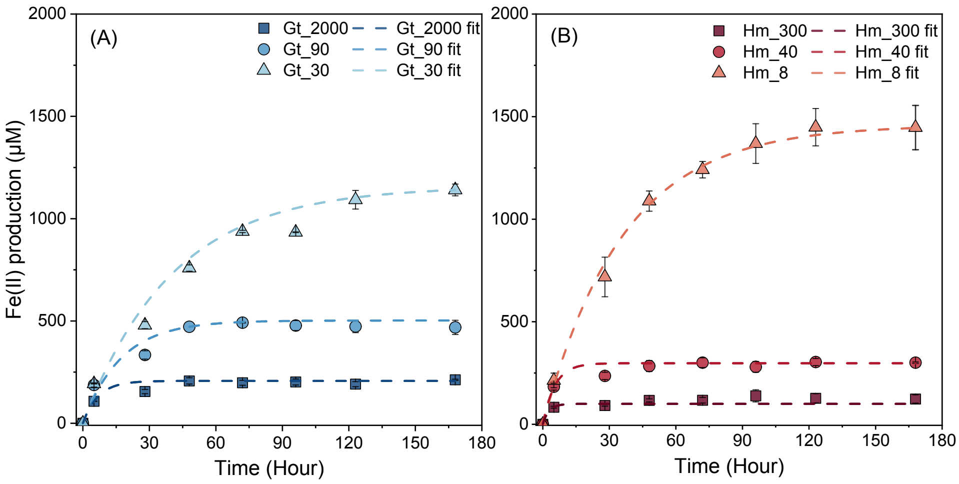

Figure S11. Total Fe(II) production in the absence of AQDS as an electron shuttle by microbial Fe(III) reduction of goethite (blue) (A) and hematite (red) (B) as a function of particle size. The dashed lines denote the fit based on pseudo-first-order kinetics. The symbols correspond to the average biological duplicates. The error bars depict the deviation of single measurements from the mean. Experimental conditions: 10 mM Fe(III) of goethite/hematite; 5×10^8 cells mL^{-1} *S. oneidensis* MR-1; pH 7.

Table S3. Reduction rate constants of goethite and hematite as a function of particle size.

Mineral	Microbial reduction in the presence of AQDS [a] h^{-1} ($\times10^{-3}$)	Microbial reduction in the absence of AQDS [b] h^{-1} ($\times10^{-3}$)
Gt_2000	1.45 ± 0.03	0.25 ± 0.02
Gt_90	2.21 ± 0.19	0.66 ± 0.01
Gt_30	6.05 ± 0.17	1.33 ± 0.01
Hm_300	0.48 ± 0.02	0.13 ± 0.01
Hm_40	1.74 ± 0.10	0.34 ± 0.03
Hm_8	6.20 ± 0.36	1.87 ± 0.05

[a] Microbial reduction in the presence of AQDS was carried out in triplicates. The values represent the mean ± standard deviation for a given mineral. [b] Microbial reduction in the absence of AQDS was conducted in duplicates. The reported rate constants are the average of the duplicate measurements, ± depict deviation of single measurements from the mean.

S4 Data collection and calculation for RDA and Pearson correlation analysis

The reduction extents and rates have been suggested to be influenced by a variety of factors including Fe(III) concentration, reactive surface area, surface charge, crystallite size, and thermodynamic properties[6-12]. In the present study, detrended correspondence analysis (DCA) and redundancy analysis (RDA) and was conducted by CANOCO 5 software (Microcomputer Power Co., USA), and Person correlation was performed with Origin 2021 (9.8) (OriginLab Co., USA), to clarify the impact of those related variables. For DCA, gradient lengths of the four axes were used to confirm the suitablity of RDA for our dataset. For RDA, we employed the default settings in CANOCO 5, specifying the response variables as the reduction extents and rates and the explanatory variables as the mineral related varibles. Pearson correlation analysis was performed in Origin 2021 using standard settings, with significance levels set at $P < 0.05$; 0.01; 0.001. Herein, the respective variables are described below and incorporated into summary tables for MER and microbial reduction experiments (Tables S4 and S5).

Particle number: We herein determined the particle number (N_c) based on added Fe concentration and assumed the shape of goethite (cuboid) and hematite (sphere), according to eq. S1.

$$N_c = \frac{C \times V_s}{fr \times r \times V_p} \quad \text{eq. S1}$$

Where C is the concentration of added Fe(III), V_s denotes the volume of solutions, f_r represents the mass fraction of Fe in respective iron (oxyhydr)oxides, r and V_p are the density and volume of respective iron (oxyhydr)oxides.

Crystallite size: We calculated the average crystallite size (D_p), based on the Scherrer equation[13], eq. S2.

$$D_p = \frac{K\lambda}{\beta cos\theta} \quad \text{eq. S2}$$

Where K is the Scherrer constant, λ represents the X-ray wavelength, β denotes FWHM (Full Width at Half Maximum) of the XRD reflections, and θ is the XRD reflections position.

***$\Delta_r G$*:** The Reaction free energies in MER experiments were calculated from differences in reduction potential between iron (oxyhydr)oxide and applied potential, according to eq. S3.

$$\Delta_r G = -nF \cdot (E_H^{oxide} - E_H^{MER}) = -nF \cdot (E_H^0 - 0.059 \log \alpha \cdot \left[Fe_{aq}^{2+}\right] - 3 \cdot 0.059 \cdot pH - E_H^{MER}) \quad \text{eq. S3}$$

Where n is the number of transferred electrons; F denotes Faraday constants; E_H^0 represents the standard reduction potentials of respective iron (oxyhydr)oxides, which are calculated according to eq. 5 and 6; 0.059 is the term RT/F at 25°C (R is the gas constant and T is the absolute temperature = 298.15 K); 3 is the molar ratio of protons to electrons in eq. S4 and S5; $\left[Fe_{aq}^{2+}\right]$is the aqueous ferrous iron concentration and and α is the activity coefficient of Fe_{aq}^{2+} calculated usingthe Davies equation[14] (0.785 based on eq. S6).

α-FeOOH + e^- + $3H^+ \rightleftharpoons Fe^{2+} + 2H_2O$ eq. S4

α-Fe_2O_3 + $2e^-$ + $6H^+ \rightleftharpoons 2Fe^{2+} + 3H_2O$ eq. S5

$$\log(\alpha) = -A \cdot Z_a \cdot Z_b \cdot \left(\frac{\sqrt{I}}{1+\sqrt{I}} - 0.3 \cdot I\right) \quad \text{eq. S6}$$

where Z_a and Z_b are the valences of an electrolyte dissociating into ions a and b, I is the ionic strength and equals 0.1 M, and A is a temperature-dependent parameter that equals 0.5 for water at 25°C.

Equation S3 shows that $\Delta_r G$ linearly correlates with logarithm of the dissolved ferrous iron concentration. As $[Fe^{2+}_{aq}]$ increased over the course of reductive process, we need to explicitly select $[Fe^{2+}_{aq}]$ for each iron (oxyhydr)oxide and individual E_H^{MER}. We herein calculated $\Delta_r G$ for selected $\{Fe^{2+}_{aq}\}$ that corresponded to the time of the maximum rate of electron transfer, identified from the maximum in the reductive current peaks.

Reactive sites: we herein calculated reactive surface sites based on the amount of adsorbed Fe(II). We determined the adsorbed phase ($Fe(II)_{ads}$) by the subtraction of measured aqueous phase ($Fe(II)_{aq}$) from the aqueous and adsorbed phase ($Fe(II)_{aq+ads}$). Different Fe(II) phases were obtained using sequential HCl extraction. An aliquot of the suspension (0.2 mL) was removed inside the N_2-filled glovebox, with the aqueous and solid phases separated by centrifugation (12100 × g, 15 min). The $Fe(II)_{aq}$ was determined from the supernatant. Another aliquot of the suspension (0.2 mL) was removed and mixed with 0.5 M HCl (30 mins at 25°C in the glovebox) to extract all the

adsorbed phase. Afterward, aqueous and solid phases were separated by centrifugation (12100 × g, 15 min) and the $Fe(II)_{aq+ads}$ were determined from the supernatant.

In addition, determining reactive surface sites also required making some assumptions about our systems: (1) In the presence of microbes and iron (oxyhydr)oxides, we assumed that dissolved Fe(II) mainly got adsorbed onto the surface of the iron (oxyhydr)oxides (2) We took the final equilibrium Fe(II) adsorption capacity as the maximum adsorption capacity. (3) We also assume that each reaction site can only bind one Fe ion. Therefore, based on Fe(II) and these assumptions, the number of reaction sites is calculated according to eq. S7

$$N_{sites} = C_{ads} \times V_s \times N_A \quad \text{eq. S7}$$

Where C_{ads} is the equilibrium concentration of $Fe(II)_{ads}$, V_s denotes the volume of incubation suspension, N_A represents Avogadro's number.

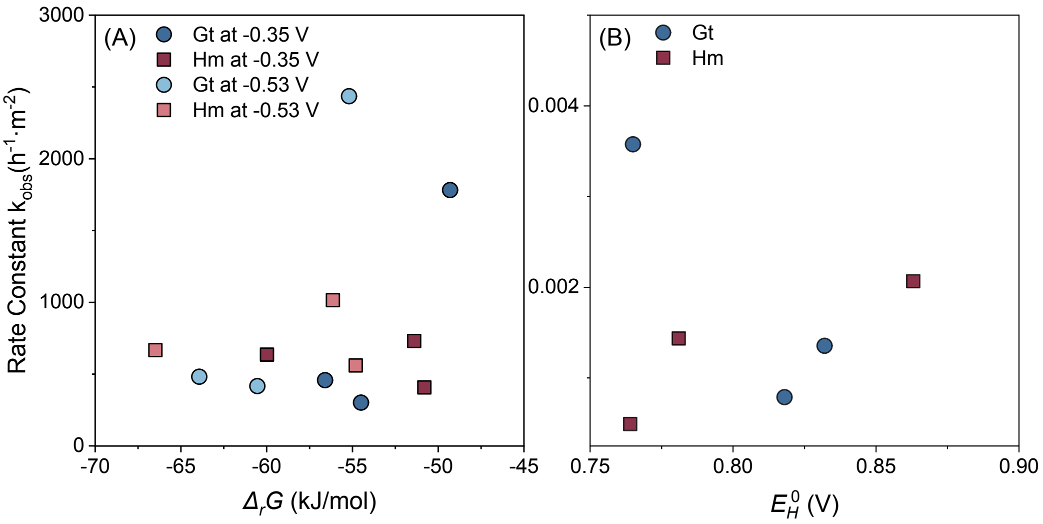

Figure S12. Correlation analysis between rate constant normalized using BET-derived surface area and free reaction energy in MER experiments (A), and standard redox potential in microbial reduction experiments (B). The data indicate that the combination of thermodynamic driving force (ΔrG) and surface area cannot account for the variations in rate constants.

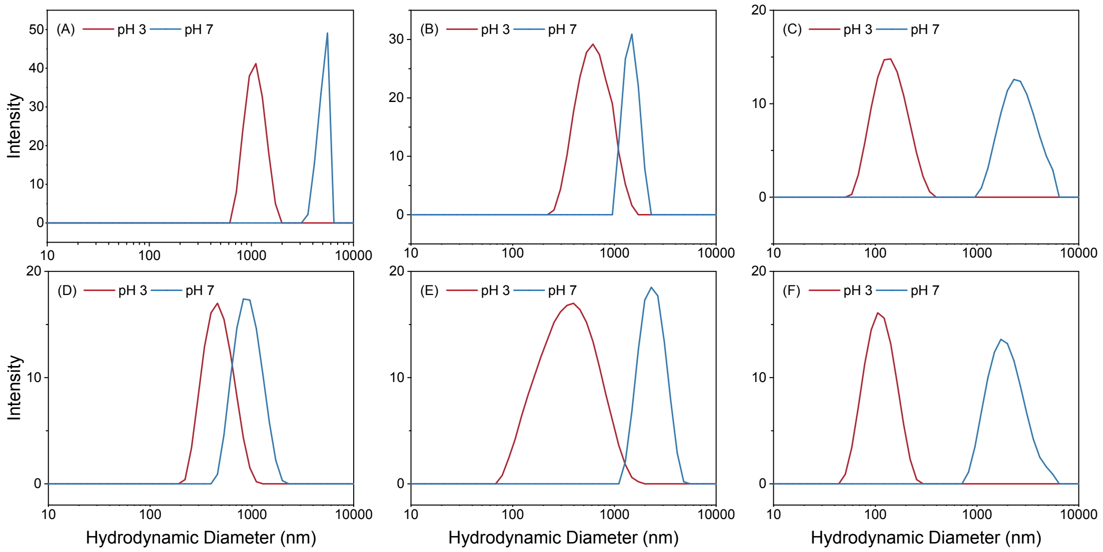

Figure S13. Hydrodynamic diameter of different-sized iron (oxyhydr)oxides at pH 3 and pH 7. Gt_2000 (A), Gt_90 (B), Gt_30 (C), Hm_300 (D), Hm_40 (E), Hm_8 (F).

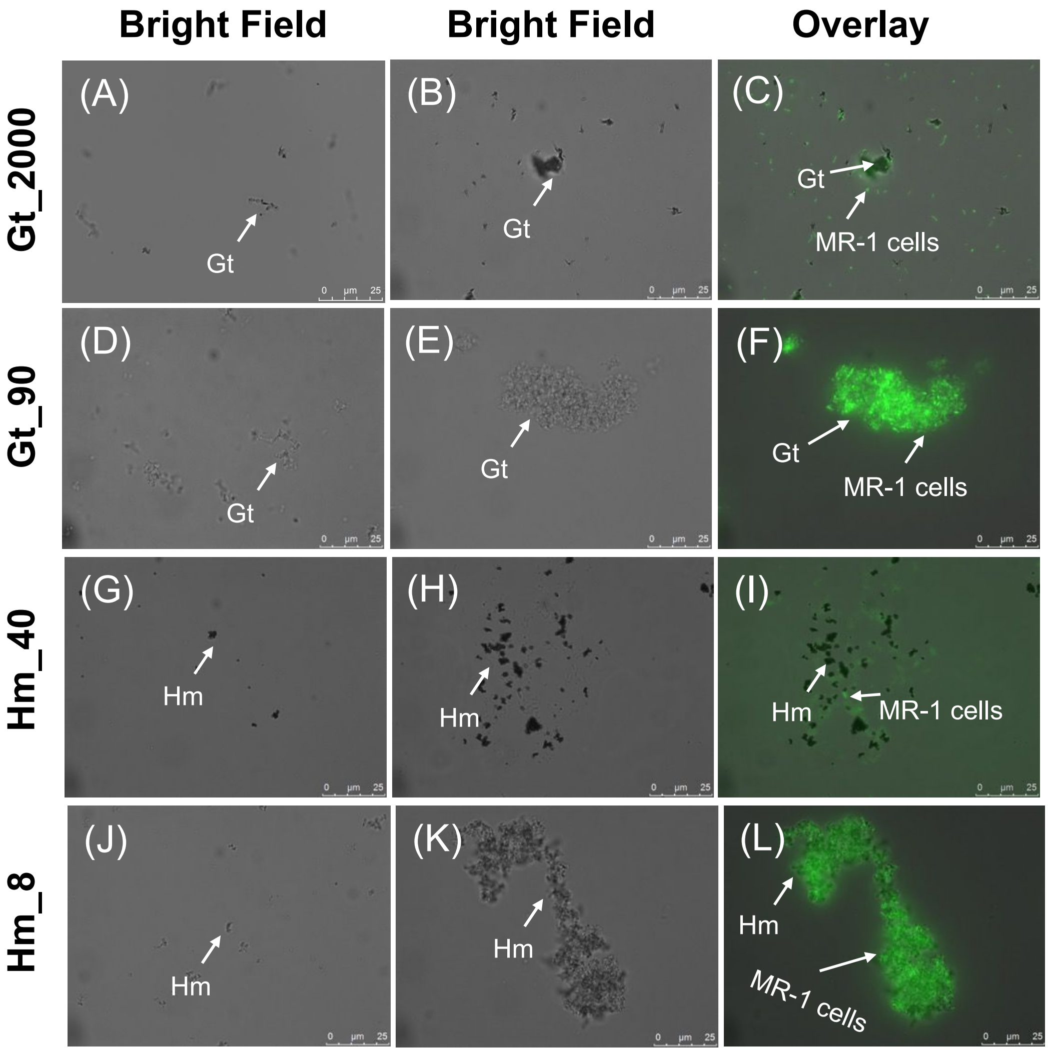

Figure S14. Aggregation of iron (oxyhydr)oxides and *S. oneidensis* MR-1 cells (stained by 4',6-diamidino-2-phenylin-dole (DAPI)) analyzed by fluorescence microscopy. Pure iron (oxyhydr)oxides are shown in bright field images (A, D, G, J), iron (oxyhydr)oxides with cells are shown in bright field images (B, E, H, K) and overlay of bright field and fluorescence images (C, F, I, L). The gray and black parts represent iron (oxyhydr)oxides, and the green parts represent MR-1 cells.

Table S4. Interrelated variables for RDA analysis obtained from mediated electrochemical reduction experiments

Samples [a]	Particle number	Surface charge	Crystallite size	$\Delta_r G$	TEM SA	BET SA	Eh
	$\times 10^8$ particles	mV	nm	KJ/mol	$\times 10^{-3}$ m^2	$\times 10^{-3}$ m^2	mV
S1	3.1	32.8	20.8	-49.3	0.5	1.6	765
S2	26920.8	33.4	11.0	-54.5	9.9	11.2	818
S3	83646.6	31.8	7.5	-56.6	11.0	17.9	832
S4	70.4	21.7	22.6	-50.8	0.6	3.9	764
S5	2750.0	29.8	17.3	-51.4	2.1	4.9	781
S6	336763.9	34.5	12.2	-60.0	12.6	12.0	863
S7	1.3	32.8	20.8	-55.2	0.5	1.6	765
S8	26920.8	33.4	11.0	-60.5	9.9	11.2	818
S9	83646.6	31.8	7.5	-63.9	11.0	17.9	832
S10	3.3	21.7	22.6	-54.8	0.6	3.9	764
S11	2750.0	29.8	17.3	-56.1	2.1	4.9	781
S12	336763.9	34.5	12.2	-66.5	12.6	12.0	863

[a] S1-S6 represent MER experiments at E_H^{MER} = -0.35V for iron oxides with different sizes: Gt_2000, Gt_90, Gt_30, Hm_300, Hm_40 and Hm_8 performed for; S7-S12 represent the MER experiments for the same set of minerals at E_H^{MER} = -0.53V.

Table S5. Interrelated variables for RDA analysis obtained from microbial Fe(III) reduction experiments.

Samples [a]	Particle number $\times 10^{10}$	Surface charge mV	Crystallite size nm	Reactive sites $\times 10^{18}$	BET SA m^2	Eh mV
S1	7.7	32.8	20.8	2.3	0.4	765
S2	67302	33.4	11.0	5.5	2.8	818
S3	209117	31.8	7.5	3.6	4.5	832
S4	90	21.7	22.6	3.1	1.0	764
S5	15455	29.8	17.3	2.8	1.2	781
S6	1462495	34.5	12.2	10.0	3.0	863
S7	3.9	32.8	20.8	1.5	0.2	765
S8	33651	33.4	11.0	4.1	1.4	818
S9	7728	29.8	17.3	2.0	0.6	781
S10	731248	34.5	12.2	9.3	1.5	863

[a] Iron (oxyhydr)oxides with different sizes, S1-S6 represent Gt_2000, Gt_90, Gt_30, Hm_300, Hm_40 and Hm_8 were performed for microbial reduction at initial Fe(III)=10 mM; S7-S10 represent Gt_2000, Gt_90, Hm_40 and Hm_8 were performed for microbial reduction at initial Fe(III)=5 mM;

S5 Calculation of the origin of reduced Fe based on their isotope concentration

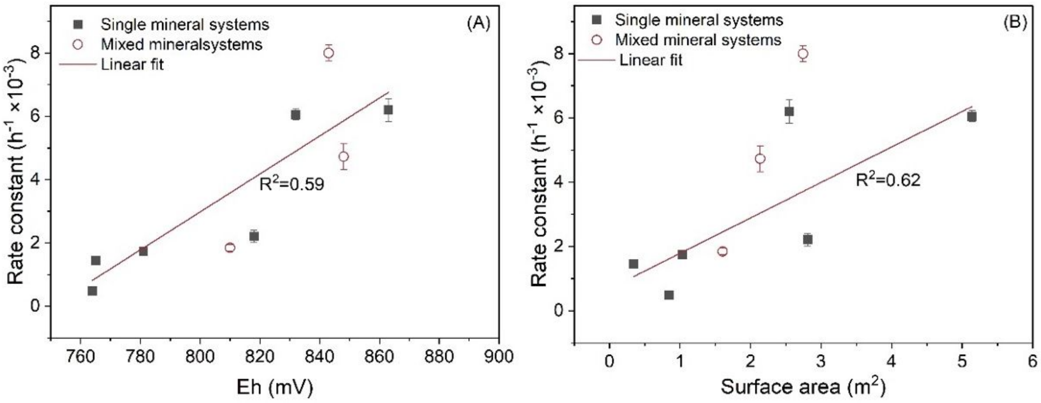

Figure S15. Linear combination between reduction rate constant and Eh (A) and surface area (B). The black marker represents Eh/SA of single mineral systems, The red marker represents Eh_{mix}/SA_{mix} of mixed mineral systems. The data indicates that neither the surface area nor reduction potential of the systems can fully account for the variations in rate constants.

A mixed potential (Eh_{mix}) can be determined by the weighted sum of Nernstian terms for each of the redox couples[15]. However, the values of exchange current density are unknown for different particle sizes. Given that surface area is a critical controlling factor for Eh, we thereby assume the overall Eh_{mix} of the systems as a surface area weighted sum of the Eh values of the individual minerals. The formula for calculating Eh_{mix} based on surface area-weighted contribution can be expressed as:

$$Eh_{mix} = \sum_{i=1}^{m} \frac{SA_i \times \mathrm{E}h_i}{\sum SA_i}$$

This approximation helps to quantify how the different minerals, with their varying particle sizes and Eh values, contribute to the system's overall redox potential. Minerals

with smaller particle sizes and higher surface areas dominate the Eh_{mix}, making this approach a suitable method for approximating the redox environment of mixed system. To investigate size-preferential microbial Fe(III) reduction in environmentally relevant systems, we designed three mixed mineral treatments (Materials and methods). The respective concentrations were chosen to approximate their number-based size distribution based on Pareto's law and the following equations,

$$\frac{C_{small\,mineral}}{C_{large\,mineral}} = \frac{M_{small} \times f_{small} \times N_{small}}{M_{large} \times f_{large} \times N_{large}} \quad \text{eq. S7}$$

Where $C_{small\,mineral}$ and $C_{large\,mineral}$ are the Fe concentrations of small and large minerals, respectively. M_{small} denotes mass of primary small mineral particles, f_{small} represents Fe mass fraction of small mineral particles, N_{small} is the particle numbers in natural environment[16, 17]. The respective concentrations of Fe in the mixed minerals are as follows (1) Gt_2000 (5 mM) and Gt_90 (5 mM), (2) Hm_40 (3.5 mM) and Hm_8 (6.5 mM), and (3) Gt_90 (4.5 mM) and Hm_8 (5.5 mM).

Here, we present in detail the calculation of the respective Fe concentrations in mixed mineral systems using the combination of Gt_2000 and Gt_90 as an example.

$$\frac{C_{Gt\_2000}}{C_{Gt\_90}} = \frac{M_{Gt\_2000} \times f_{Gt\_2000} \times N_{Gt\_2000}}{M_{Gt\_90} \times f_{Gt\_90} \times N_{Gt\_90}}$$

$$\frac{C_{Gt\_2000}}{C_{Gt\_90}} = \frac{\rho_{Gt} \times V_{Gt\_2000} \times f_{Gt\_2000}}{\rho_{Gt} \times V_{Gt\_90} \times f_{Gt\_90}} \times \frac{N_{Gt\_2000}}{N_{Gt\_90}}$$

We then assumed goethite morphology as an ideal rectangle, and took the average length and width from TEM images (statically above 100 particles) as length and width of the ideal rectangle, f_{Gt_2000} and f_{Gt_90} represent Fe mass fraction in Gt_2000 and

Gt_90 respectively, $\frac{N_{Gt\_2000}}{N_{Gt\_90}}$ denotes the particle number ratio in natural environments based on Pareto's law (Ranville and Montano, 2015; Westerhoff et al., 2018).

$$\frac{C_{Gt\_2000}}{C_{Gt\_90}} = \frac{L_{Gt\_2000} \times W_{Gt\_2000} \times W_{Gt\_2000}}{L_{Gt\_90} \times W_{Gt\_90} \times W_{Gt\_90}} \times \frac{N_{Gt\_2000}}{N_{Gt\_90}}$$

$$\frac{C_{Gt\_2000}}{C_{Gt\_90}} = \frac{L_{Gt\_2000} \times W_{Gt\_2000} \times W_{Gt\_2000}}{L_{Gt\_90} \times W_{Gt\_90} \times W_{Gt\_90}} \times \frac{N_{Gt\_2000}}{N_{Gt\_90}}$$

$$\frac{C_{Gt\_2000}}{C_{Gt\_90}} = \frac{2000 \times 200 \times 200}{100 \times 10 \times 10} \times \frac{1}{8000} = \frac{1}{1}$$

In order to keep the initial Fe concentration constant, we herein set the total Fe concentration to be the same as in the single mineral systems, thereby the concentration of Gt_2000 and Gt_90 were 5 mM and 5 mM respectively.

To track the origin of reduced iron in the mixture of ^{NA}mineral and ^{56}mineral, we calculated the amount of Fe(II) from corresponding minerals based on Fe isotopic distribution. The specific calculation equations were shown in eq. S8-9.

$$C_{NAFe\ mineral} = \frac{C_{54Fe(II)}}{M_{54Fe} \times f_{54Fe}} \quad \text{eq. S8}$$

$$C_{56Fe\ mineral} = \frac{C_{56Fe(II)} - C_{NAFe\ mineral} \times f_{56Fe}}{M_{56Fe}} \quad \text{eq. S9}$$

Where $C_{NAFe\ mineral}$ and $C_{56Fe\ mineral}$ are the concentration of reduced ^{NA}Fe and ^{56}Fe synthesized minerals, $C_{54Fe(II)}$ and $C_{56Fe(II)}$ denotes the isotopic concentration of Fe(II), M_{54Fe} and M_{56Fe} represent the molar mass of the respective iron isotopes, f_{54Fe} and f_{56Fe} are the fraction of the respective iron isotope.

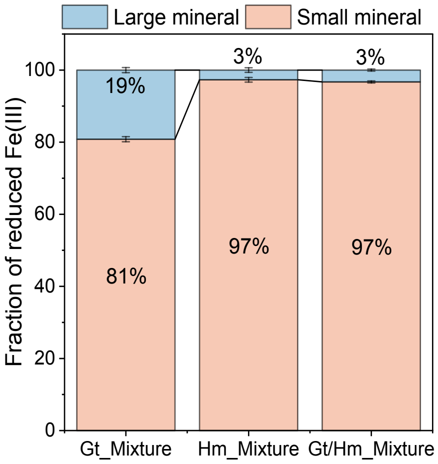

Figure S16. Fraction of Fe(II) derived from specific-sized minerals in the mixed mineral systems. Large mineral represents Gt_2000, Hm_40, and Gt_90 in the goethite, hematite and goethite/hematite mixed minerals systems, respectively. Small mineral represents Gt_90, Hm_8, and Hm_8 in the goethite, hematite and goethite/hematite mixed minerals systems, respectively.

Table S6. Reduction extents and rate constants in mixed mineral system.

Mineral mixed systems	Minerals	Extents	Rate constants
		%	1/h ($\times 10^{-3}$)
Gt mixed [a]	Gt_2000	2.1± 0.2	0.4 ± 0.0
	Gt_90	11.2 ± 1.3	2.4 ± 0.4
Hm mixed [b]	Hm_40	1.2 ± 0.1	0.4 ± 0.1
	Hm_8	22.5 ± 0.4	7.9 ± 0.8
Gt/Hm mixed [c]	Gt_90	2.0 ± 0.2	0.5 ± 0.1
	Hm_8	45.6 ± 3.6	11.1 ± 2.2

Microbial Fe(III) reduction of mineral mixed systems were carried out in triplicates, the values represent the mean ± standard deviation for the specific mineral. [a] goethite mineral mixed system containing 5 mM ^{56}Gt_2000 & 5 mM ^{NA}Gt_90. [b] hematite mineral mixed system containing 3.5 mM ^{56}Hm_40 & 6.5 mM ^{NA}Hm_8. [c] goethite and hematite mineral mixed system containing 4.5 mM ^{NA}Gt_90 & 5.5 mM ^{56}Hm_8.

S6 Possible mechanisms for preferential microbial reduction

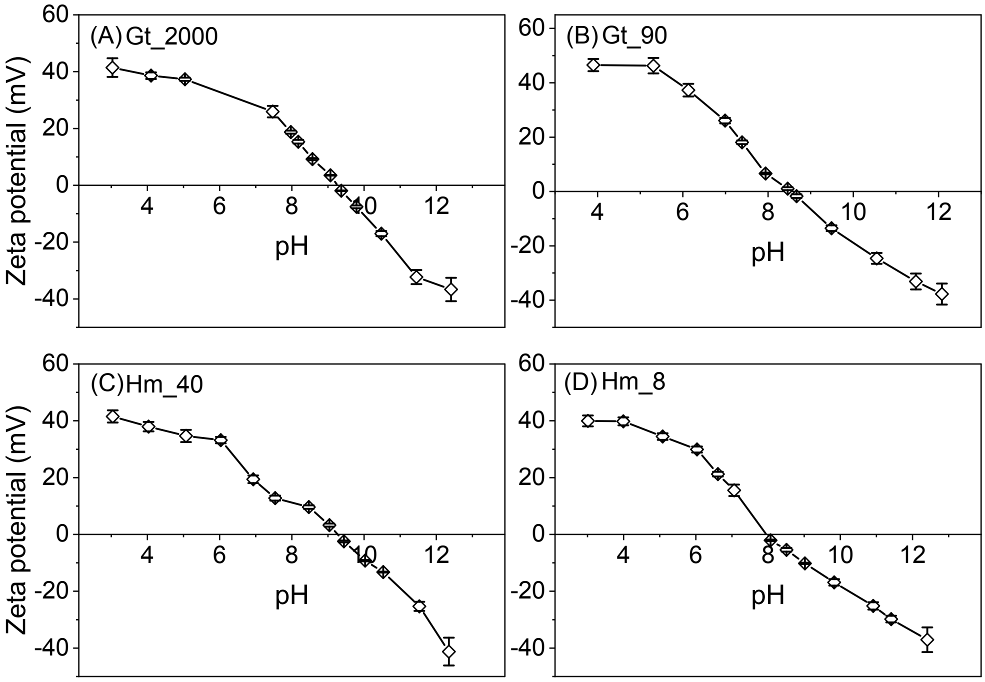

Figure S17. Measured Zeta potentials for Gt_2000 (A), Gt_90 (B), Hm_40 (C), Hm_8 (D) as a function of pH. Measurements were performed in triplicate; error bars represent standard deviation. pH was manually adjusted by adding NaOH and HCl aqueous solutions (10 mM and 100 mM) and was measured using a pH meter.

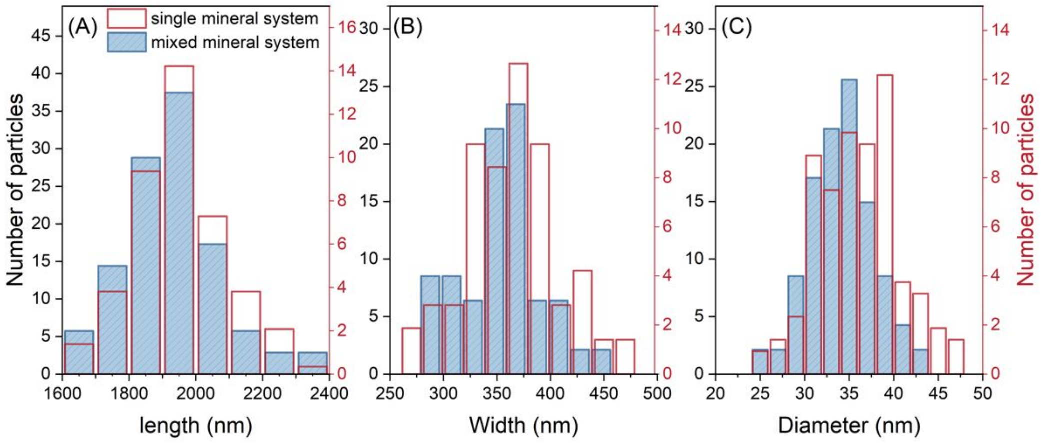

Figure S18. Particle size distribution of goethite and hematite in single and mixed mineral systems. Blue columns represent particle size in mixed mineral systems, while red columns represent particle size in single mineral systems after 14 days. Length and width of Gt_2000 (A, B), diameter of Hm_40 (C). The results suggest that, based on the Kruskal-Wallis test, there are no statistically significant differences ($P > 0.05$), in

the particle size of minerals between mixed-mineral systems and single-mineral systems.

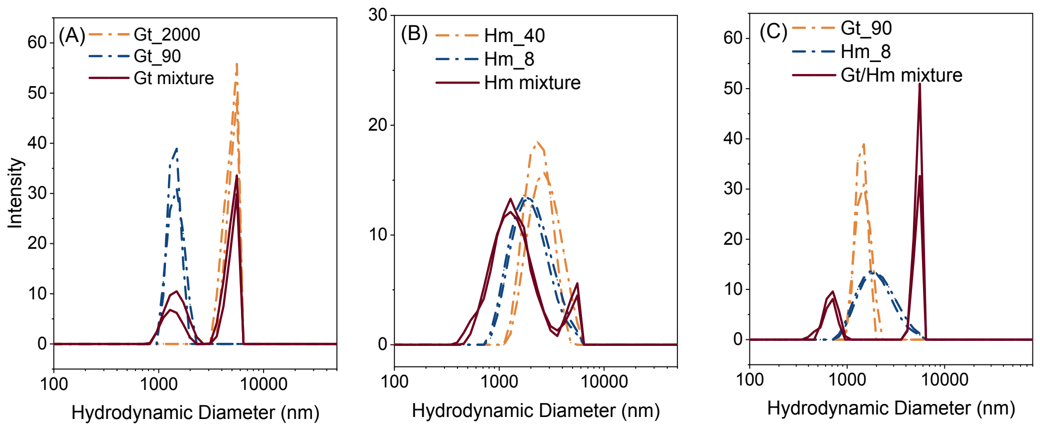

Figure S19. Measured aggregation size of iron oxides in single/mixed mineral systems. Gt_2000 & Gt_90 (A), Hm_40 & Hm_8 (B), Gt_90 & Hm 8 (C). The measurements were carried out in duplicate. Suspension was vortexed for 30 seconds followed by sitting for 20 seconds to allow any extremely large aggregates to settle out of solutions.

Table S7. Hydrodynamic diameter in mixed mineral systems

Gt_2000 & Gt_90		Hm_40 & Hm_8		Gt_90 & Hm_8	
Peak 1	Peak 2	Peak 1	Peak 2	Peak 1	Peak 2
nm	nm	nm	nm	nm	nm
1384	5237	1434	5134	701	5334
1502	5313	1718	5260	734	5197

Mineral mixtures tend to result in heteroaggregation due to their morphological variations and surface charge disequilibrium[18-20]. Aggregation can impact the accessibility of reactive surface sites, depending on the extent of aggregation and the shape of the aggregates (i.e., loose versus compact packing). In our present study, two types of behavior can be observed in the three mixed mineral systems. For (1) Hm_8 &

Hm_40 and (2) Hm_8 & Gt_90 mixtures, two peaks corresponding to small aggregates and large aggregates could be discerned (SI, Fig. S13). The large aggregates exhibited a larger size in the mixed system compared to the Hm_40 and Gt_90 single system, indicative of heteroaggregation. This indicates higher aggregation extents that could mask more reactive sites and suppress reduction. Overall, heteroaggregation could simultaneously decrease the reduction of larger minerals and promote the reduction of smaller-sized minerals in the Hm_8 & Hm_40 and Hm_8 & Gt_90 mixtures.

In the second behavior, for Gt_90 & Gt_2000 mixture, a shift towards larger aggregate size indicative of heteroaggregation was not observed (SI, Fig. S13). This could be explained by the different morphology and aggregation patterns of goethite. This suggests that heteroaggregation played a minimal role in affecting reduction rates in this system.

To evaluate how aggregate pore throat can impact AQDS-mediated reduction by *Shewanella*, we estimated the aggregate pore throat assuming close-packed sphere configuration using the following formula:

$R_{pore}=(2\sqrt{3}/3-1)R_{particle}$

We obtained values of 23 nm for Hm_300, 3 nm for Hm_40 and 0.6 nm for Hm_8. The size of an AQDS molecule is 0.5-1.5 nm. Hence, AQDS diffusion into Hm_8 aggregates could be significantly slowed. However, previous studies on ~5 nm ferrihydrite have shown intra-aggregate pores with an average size around 2 nm, suggesting that smaller primary particle tends to form more compact aggregates.

Furthermore, our experiments showed that Hm_8 was reduced the fastest out of all the minerals tested. Hence, we conclude that AQDS diffusion was not limited by the aggregate pore throat.

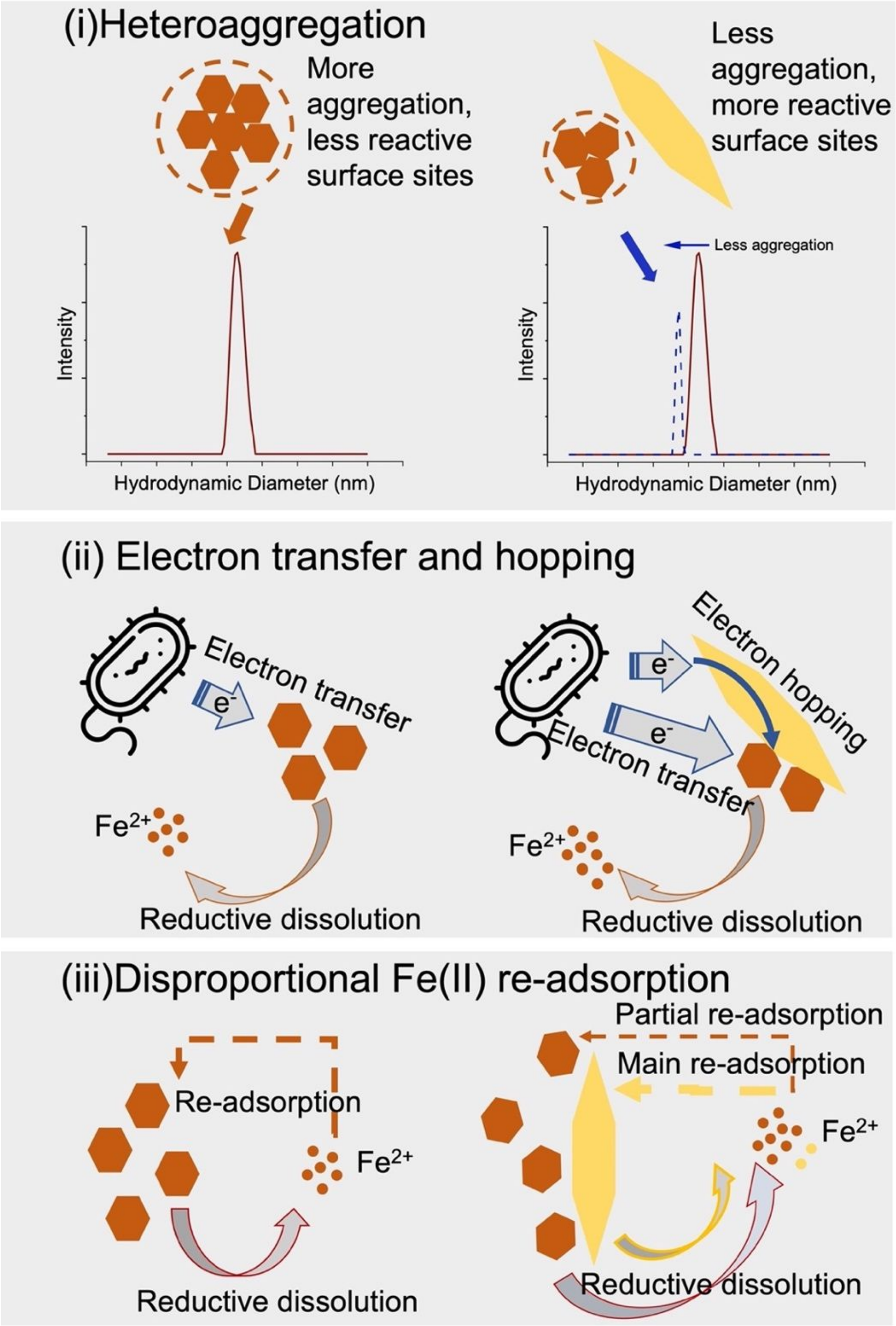

Figure S20. Schematic figure of potential mechanisms that explain the promoted reduction of small-sized particles, and the suppressed reduction of large-sized particles in mixed mineral systems compared to single mineral systems

S7 Environmental implications

Reduction extents of iron (oxyhydr)oxides are highly size-dependent, which has significant implications for natural aquatic systems. In aquatic systems, the particle size distribution and their relative abundance typically follow Pareto's law, meaning that smaller particles tend to be more abundant. To illustrate the impact of particle size on the reducible phase, we developed a model assuming a total Fe concentration of 10 mM, with particle sizes distributed across three ranges: 1–10 nm, 10–100 nm, and >100 nm. We assumed that Gt_20 and Hm_8 represent particles within the 0-10 nm size range; Gt_90 and Hm_40 represent particles across 10-100 nm range; and Gt_2000 and Hm_300 represent particles > 100 nm.

Using our experimental data, which approximately quantifies the reduction extent for each size range, we calculated the contribution of each size fraction to the total reducible Fe content. When particle size effects are considered, the reducible Fe content is 10% for goethite and 12% for hematite. However, if size effects are ignored, the reducible Fe content drops significantly to 2% for goethite and 5% for hematite. Furthermore, when considering only particles larger than 0.45 μm—which is the typical size separated in field filtration, the reducible Fe content falls dramatically to just 0.7% for goethite and 1.7% for hematite. These results demonstrate that traditional filtration methods can lead to a significant underestimation of the reducible solid phase in natural systems. By excluding the smaller, more reactive nanoparticles, standard filtration approaches fail to capture the full extent of Fe reduction potential.

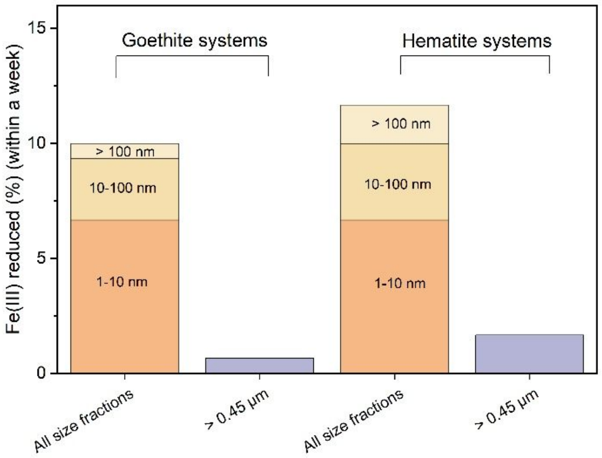

Figure S21. Modeled distribution of reducible Fe(III) fraction across all different particle size ranges (1-10 nm, 10-100 nm, >100 nm), or particles larger than 0.45 μm for goethite and hematite. The model illustrates the relative contribution of each size fraction to the total reducible Fe(III) pool, highlighting the impact of particle size on reduction behavior and the substantial underestimation of reducible Fe if particles smaller than 0.45 μm are neglected.

References

1. Anschutz, A. J.; Penn, R. L., Reduction of crystalline iron (III) oxyhydroxides using hydroquinone: Influence of phase and particle size. *Geochem. Trans.* **2005,** *6* (3), 1-7.
2. Madden, A. S.; Hochella Jr, M. F.; Luxton, T. P., Insights for size-dependent reactivity of hematite nanomineral surfaces through Cu2+ sorption. *Geochim. Cosmochim. Acta* **2006,** *70* (16), 4095-4104.
3. Schwertmann, U.; Cornell, R. M., *Iron oxides in the laboratory: preparation and characterization*. John Wiley & Sons: 2008.
4. Lagarec, K.; Rancourt, D., Extended Voigt-based analytic lineshape method for determining N-dimensional correlated hyperfine parameter distributions in Mössbauer spectroscopy. *Nucl. Instrum. Meth. B.* **1997,** *129* (2), 266-280.
5. Lowry, G. V.; Hill, R. J.; Harper, S.; Rawle, A. F.; Hendren, C. O.; Klaessig, F.; Nobbmann, U.; Sayre, P.; Rumble, J., Guidance to improve the scientific value of zeta-potential measurements in nanoEHS. *Environ. Sci. Nano* **2016,** *3* (5), 953-965.
6. Roden, E. E.; Zachara, J. M., Microbial reduction of crystalline iron (III) oxides: influence of oxide surface area and potential for cell growth. *Environ. Sci. Technol.* **1996,** *30* (5), 1618-1628.
7. Roden, E. E., Fe (III) oxide reactivity toward biological versus chemical reduction. *Environ. Sci. Technol.* **2003,** *37* (7), 1319-1324.
8. O'Loughlin, E. J., Effects of electron transfer mediators on the bioreduction of lepidocrocite (γ-FeOOH) by Shewanella putrefaciens CN32. *Environ. Sci. Technol.* **2008,** *42* (18), 6876-6882.
9. Bose, S.; Hochella Jr, M. F.; Gorby, Y. A.; Kennedy, D. W.; McCready, D. E.; Madden, A. S.; Lower, B. H., Bioreduction of hematite nanoparticles by the dissimilatory iron reducing bacterium Shewanella oneidensis MR-1. *Geochim. Cosmochim. Acta* **2009,** *73* (4), 962-976.
10. Cutting, R.; Coker, V.; Fellowes, J.; Lloyd, J.; Vaughan, D., Mineralogical and morphological constraints on the reduction of Fe (III) minerals by Geobacter sulfurreducens. *Geochim. Cosmochim. Acta* **2009,** *73* (14), 4004-4022.
11. Echigo, T.; Aruguete, D. M.; Murayama, M.; Hochella Jr, M. F., Influence of size, morphology, surface structure, and aggregation state on reductive dissolution of hematite nanoparticles with ascorbic acid. *Geochim. Cosmochim. Acta* **2012,** *90*, 149-162.
12. Liu, J.; Pearce, C. I.; Shi, L.; Wang, Z.; Shi, Z.; Arenholz, E.; Rosso, K. M., Particle size effect and the mechanism of hematite reduction by the outer membrane cytochrome OmcA of Shewanella oneidensis MR-1. *Geochim. Cosmochim. Acta* **2016,** *193*, 160-175.
13. Patterson, A., The Scherrer formula for X-ray particle size determination. *Phys. Rev.* **1939,** *56* (10), 978.

14. Davies, C. W., 397. The extent of dissociation of salts in water. Part VIII. An equation for the mean ionic activity coefficient of an electrolyte in water, and a revision of the dissociation constants of some sulphates. *J. Chem. Soc. (Resumed)* **1938**, 2093-2098.
15. Shi, Z.; Nurmi, J. T.; Tratnyek, P. G., Effects of nano zero-valent iron on oxidation− reduction potential. *Environ. Sci. Technol.* **2011,** *45* (4), 1586-1592.
16. Ranville, J.; Montano, M. D., Size distributions. In *Frontiers of Nanoscience*, Elsevier: 2015; Vol. 8, pp 91-121.
17. Westerhoff, P.; Atkinson, A.; Fortner, J.; Wong, M. S.; Zimmerman, J.; Gardea-Torresdey, J.; Ranville, J.; Herckes, P., Low risk posed by engineered and incidental nanoparticles in drinking water. *Nat. Nanotechnol.* **2018,** *13* (8), 661-669.
18. Ma, J.; Jing, Y.; Gao, L.; Chen, J.; Wang, Z.; Weng, L.; Li, H.; Chen, Y.; Li, Y., Hetero-aggregation of goethite and ferrihydrite nanoparticles controlled by goethite nanoparticles with elongated morphology. *Sci. Total Environ.* **2020,** *748*, 141536.
19. Gupta, G. S.; Senapati, V. A.; Dhawan, A.; Shanker, R., Heteroagglomeration of zinc oxide nanoparticles with clay mineral modulates the bioavailability and toxicity of nanoparticle in Tetrahymena pyriformis. *J. Colloid Interface Sci.* **2017,** *495*, 9-18.
20. Wang, D.; Jin, Y.; Jaisi, D. P., Effect of size-selective retention on the cotransport of hydroxyapatite and goethite nanoparticles in saturated porous media. *Environ. Sci. Technol.* **2015,** *49* (14), 8461-8470.
